# Anemia among Syrian Refugee Children Aged 6 to 23 Months Living in Greater Beirut, Lebanon, including the Voices of Mothers’ and Local Healthcare Staff: A Mixed-Methods Study

**DOI:** 10.3390/nu15030700

**Published:** 2023-01-30

**Authors:** Theresa Jeremias, Joana Abou-Rizk, Leonie Burgard, Isa Entenmann, Lara Nasreddine, Lamis Jomaa, Nahla Hwalla, Jan Frank, Veronika Scherbaum

**Affiliations:** 1 Department of Food Biofunctionality (140b), Institute of Nutritional Sciences, University of Hohenheim, 70599 Stuttgart, Germany; 2 Department of Nutrition and Food Sciences, American University of Beirut, Beirut 11-0236, Lebanon; 3 Department of Human Sciences, College of Health and Sciences, North Carolina Central University, Durham, NC 27707, USA; 4 Faculty of Agricultural and Food Sciences, American University of Beirut, Beirut 11-0236, Lebanon

**Keywords:** anemia, complementary feeding, children aged 6 to 23 months, iron supplementation, knowledge, attitudes and perceptions, mixed-methods, Syrian refugees, Lebanon

## Abstract

Globally, the prevalence of anemia among children during the period of complementary feeding is high. A cross-sectional, mixed-methods study was conducted to examine the main determinants of anemia among Syrian refugee children aged 6 to 23 months (*n* = 215) and to illuminate the knowledge, attitudes and perceptions of their mothers and Lebanese healthcare staff on its causes and available treatment options. 42% of the children and 20% of their mothers were anemic. Determinants of child anemia were the mother having anemia or not knowing that fish/seafood is a source of iron; the child having been acutely ill the last two weeks or receiving cow’s milk, but not consuming iron-rich infant formula, added fats/oils, or fruits in the previous 24 hours. Several Syrian mothers knew some causes of anemia and named dizziness as a leading symptom but did not mention flesh foods as a key source of heme iron. They reported financial constraints in accessing iron-rich foods and supplements. Lebanese doctors largely gave appropriate dietary advice and prescribed iron supplements as treatment. Multisectoral interventions are needed that combine medical and financial support with nutrition counseling for mothers to reduce the high burden of anemia among young children living in a multiple crises situation.

## 1. Introduction

The worldwide prevalence of anemia among children aged 6 to 59 months and among pregnant women aged 15 to 49 years were estimated to be 40% and 36%, respectively. Central Asia, Middle East, and North Africa region had a predicted prevalence of anemia among young children of 33%, of which 20% was attributed to mild and 13% to moderate anemia [1].

The World Health Organization (WHO) states that anemia is a condition whereby the number of red blood cells or the hemoglobin concentration is reduced below the individual’s normal level with different cut-off points for total, mild and moderate anemia [2]. Determinants of anemia in the first 1000 days of a child include: the mother having anemia during pregnancy, low birth weight of the child, the child suffering from severe infectious diseases, delayed introduction of complementary foods, not feeding foods that are rich in iron and Vitamin B12 as well as introducing foods too early that hamper iron absorption, for instance, giving cow’s milk to the child before the age of 1 year [2,3,4,5].

In 2019, according to WHO’s Global Health Observatory, the prevalence of anemia among children aged 0 to 59 months was 23% in Lebanon and 33% in the Syrian Arab Republic [6]. Evidence on the prevalence of anemia and its determinants among young Syrian children living in Syria remains scarce due to the ongoing crisis. Humanitarian actors primarily measure acute and chronic malnutrition and not anemia for various reasons [7]. Anemia is linked to child mortality through a higher vulnerability to infection and malnutrition which refugee children are more susceptible to [8]. A study from 1997 which was conducted among Palestinian refugee children aged 6 to 35 months living in Syria, Lebanon, or Gaza, found that current episodes of acute illness were a determinant of anemia among children [9]. According to the United Nations Vulnerable Assessment of Syrian Refugees in Lebanon report (2018), the majority of Syrian children under 2 years of age had suffered from a fever and half of them from diarrhea in the 2 weeks prior to the survey, resulting in a quarter of children requiring a doctor’s consultation [10]. In 2021, a national representative survey found the prevalence of anemia among Syrian refugee children aged 6 to 59 months living in Beirut, Lebanon to be 27% and that of children suffering from an acute illness to be 30%; however, the determinants of child anemia were not ascertained [11].

In 2017, Lebanon hosted 1.5 million Syrian refugees making it the country with the largest concentration of refugees per capita worldwide. Two-thirds of Syrian refugee households were found to live below the poverty line and one-third of households in Lebanon were moderately to severely food insecure [12]. Existing literature demonstrated that low socio-economic status is associated with a higher burden of anemia among children aged 6 to 59 months [13,14,15,16,17]. In our study conducted in Lebanon in 2018, we found that Syrian refugee children aged 0 to 59 months had two times the odds of being anemic, when the mother was anemic, too. Further, the diet of these Syrian refugee mothers was marked by a high intake of total fat and inadequate intakes of protein from animal-source foods that are rich in micronutrients such as iron and Vitamin B12. [18]

The underlying food insecurity in Lebanon might have compromised the quality of the Syrian mothers’ diet according to recent evidence from the authors of this study [19,20]. Further, a study in Jordan showed an association between maternal educational level and hemoglobin levels among pre-school children aged 36 to 59 months and found their diet to be low in foods that enhance iron absorption but high in foods that inhibit iron absorption [21]. A lack of knowledge of mothers on an adequate, nutrient-rich diet for themselves and their offspring can be an important contributor to child anemia [22,23,24]. According to a study undertaken in Beirut, Lebanon (2016), the majority of Syrian refuge women were not knowledgeable about maternal nutrition, half of the women had inadequate dietary practices during pregnancy and a quarter of the women had a negative attitude towards antenatal care [25].

The aim of the study was to understand the key determinants and contextual factors that might have contributed to anemia among Syrian refugee children in the complementary feeding age period. The objectives were as follows:

1. to specify the prevalence of anemia among Syrian refugee children aged 6 to 23 months living in a protracted crisis situation;
2. to identify dietary and socio-economic determinants of anemia and its moderate and mild forms among those children; and
3. to elucidate the knowledge, attitudes and perceptions of Syrian mothers and Lebanese healthcare staff on anemia and the available treatment options.

## 2. Materials and Methods

### 2.1. Study Design and Sampling Method

#### 2.1.1. Study Design

In this study, 215 Syrian refugee children aged 6 to 23 months and their mothers were included in the quantitative assessment. In the associated qualitative assessment, we included 43 Syrian mothers and 4 Lebanese healthcare staff. Details are described in Section 2.1.2 and Section 2.8.1. The main, cross-sectional study was conducted among Syrian refugee mothers and children aged 0 to 59 months visiting primary health care centers in the Greater Beirut area of Lebanon from July to October 2018 [18]. Survey sites were selected with the support of the Ministry of Public Health (MoPH), Lebanon, using high vulnerability criteria of the United Nations High Commissioner for Refugees (UNHCR) [26]. Following communities were included: Baouchriyeh, Bourj Barajneh, Bourj Hammoud, Chiyah, Mazraa, and Mousaytbeh. Hemoglobin finger pricks and anthropometric measurements of the mother and index child were assessed. A multi-component questionnaire on socio-economic status and dietary intake was applied. More details on the design of the quantitative main study, data collection and the recruitment strategy of study participants can be found in the paper from Abou-Rizk et al. (2021) [18].

The qualitative main study encompassed 11 Focus Group Discussions (FGD) with 43 Syrian mothers and female family members as well as 21 Key Informant Interviews (KII) with Lebanese health staff, staff of the UN organizations (UNO) and non-governmental organizations (NGO).

#### 2.1.2. Sampling Method

The calculation for the sample size was based on previous estimates of anemia rates among Syrian refugee children aged 6 to 59 months in Lebanon by Hossain et al. (2013–2014) [27]. The prevalence of 21% was used to calculate the sample size of 255 required study participants (power = 0.80, alpha = 0.05, and margin of error = 0.05). Accounting for a 15% non-response rate and a 10% drop-out rate a required sample size of 319 children was estimated. This sample size was achieved in the main study (*n* = 319, children aged 6 to 59 months) [18]. The prevalence of anemia among the Syrian children aged 6 to 23 months determined by a hemoglobin (Hb) level < 11 g/dL was defined as the primary outcome for this study [28]. For the objectives of this study, all young children aged 6 to 23 months and their mothers were purposefully selected from the main study (*n* = 215).

### 2.2. Anthropometric Assessment

Anthropometric variables of the child (weight, length or height) were assessed with SECA measurement equipment following standard protocols of the WHO [29]. The nutritional status of children was defined according to the WHO 2006 child growth standards [30]. Undernutrition was specified as stunting (length/height-for-age z-scores < −2), underweight (weight-for-age z-scores < −2) and wasting (weight-for-height z-scores < −2, body mass index (BMI)-for-age z-scores < −2). Overweight including obesity was defined as BMI-for-age z-scores > +2. The WHO Anthro Survey Analyzer macro for SPSS was used to derive all z-scores [31]. Outliers were identified as z-scores < −6, double-checked using box plot graphs and deleted prior to analysis.

### 2.3. Biochemical Assessment

Hemoglobin levels of the Syrian children and their mothers were assessed using finger pricks taken by certified research assistants and analyzed onsite using the ‘HemoCue Hb301 System’ device [32]. Anemia status was defined using WHO cut-off points at sea level: Hb < 11.0 g/dL for children (6 to 23 months) and pregnant mothers and Hb < 12.0 g/dL for non-pregnant mothers (irrespective of lactating status).

For children (6 to 23 months) and pregnant mothers, mild, moderate and severe anemia were defined as Hb = 10.0–10.9 g/dL, Hb = 7.0–9.9 g/dL and Hb < 7.0 g/dL, respectively. For non-pregnant mothers (irrespective of lactating status) mild, moderate and severe anemia was defined as Hb = 11.0–11.9 g/dL, Hb = 8.0–10.9 g/dL and Hb < 8.0 g/dL, respectively [28].

### 2.4. Assessment of Dietary Intake, Birth Outcomes and Health Status

#### 2.4.1. Dietary Intake

The dietary intakes of the children and mothers were collected using the quantitative 5-step multiple-pass 24 h dietary recall (24-h DR) method by the United States Department of Agriculture (USDA) [33]. The 24-h DR data were entered into NutriSurvey 2007 software with the help of the USDA food database (SR 28, version: May 2016) and a food composition table containing Lebanese foods and dishes with standardized portion sizes [34,35]. The standard food groups according to the WHO and the Food and Agriculture Organization of the United Nations (FAO) guidelines for assessing the dietary diversity of children aged 6 to 23 months were used [36,37,38]. Additional food groups were developed to explore associations between dietary intakes and all classifications of anemia (see Supplementary Table S1 for a definition of key foods and food groups).

#### 2.4.2. Birth Outcomes

The timing of breastfeeding initiation at birth was defined according to the WHO definition of early initiation of breastfeeding (within one hour after birth) [38]. The gestational age of the child was defined as ‘term ≥ 9 months’ and ’pre-term < 9 months’ serving as a proxy to the 39 weeks (8.97 months) before that WHO suggests that a Cesarean section should not be performed [39]. Categories for perceived birth weight were established as follows: low birth weight (<2.5 kg), normal birth weight (2.5–4.2 kg) and high birth weight or macrosomia (>4.2 kg) [40,41]. Breastfeeding initiation, gestational age, birth weight and length were self-reported by the mothers in retrospective.

#### 2.4.3. Health Status

Symptoms of acute and chronic illnesses, the use of standard medicine and the intake of nutrient supplements of children aged 6 to 23 months and their mothers were collected using a multiple answers questionnaire format.

### 2.5. Quantitative Assessment of Maternal Knowledge

Quantitative assessment of the mothers’ knowledge of the causes and symptoms of anemia and the dietary sources of iron were ascertained by using a multiple answers questionnaire format. Similarly, the main sources of health and nutrition messages of Syrian mothers were assessed using questions with multiple answers.

### 2.6. Food Security Status

The food security status of the Syrian mothers was measured using the individual 8-item Food Insecurity Experience Scale (FIES) by the FAO [42]. For the sake of the multiple logistic regression analyses used in this work, the FIES data were categorized into two groups (1 = food secure, 2 = food insecure) using the probability of the mother being food insecure as a basis. Details on the methodology and analysis of the full FIES data from the main study were published elsewhere [19].

### 2.7. Statistical Analysis

Quantitative data analysis was performed using the Statistical Package for Social Sciences, version 27.0 (SPSS Inc., Chicago, IL, USA) [43]. Nominal variables are expressed in *n* (%) and numerical variables in mean (±SD). Descriptive statistics, chi-square and Fisher’s exact tests were used to test the significance of associations between categorical exposure variables. One-way ANOVA was used to test the significance between means of continuous variables. Significant levels were defined as *p* < 0.05, *p* < 0.01 and *p* < 0.001.

For logistic regression analyses, the dependent, outcome variable “child anemia” was coded in a binary manner (not anemic = 0, anemic = 1). Possible determinants (socio-economic status, pregnancy outcomes, maternal and child health status, dietary intake of children) were tested using an analytical framework approach (Supplementary Figure S1). Variables that reached significance at *p* < 0.2 in the simple regression were tested for significance at *p* < 0.05 level in the multiple logistic regression models using the “Enter method”. The perceived gestational age and sex of the child were included as a priori confounders in all models. Multinominal logistic regression was used to assess the determinants of moderate and mild anemia (not anemic = 0, mildly anemic = 1, moderately anemic = 2). All significant determinants (*p* < 0.05) that were found in the multiple logistic regression analyses, were tested in the multinominal regression models.

Multicollinearity between independent variables was tested using the condition index, correlation coefficient, tolerance and variance inflation factors. The Nagel–Kerke (NK) Pseudo-R^2^, the overall percentage (>70%) and the Hosmer–Lemeshow Test were used to judge the significance and goodness-of-fit for all models. For the multinominal regression model, the McFadden (MF) Pseudo-R^2^ was used additionally. Model 1 had an NK Pseudo-R^2^ of 0.419. The multinominal regression model had a Pseudo-R^2^ of 0.388 and MF Pseudo-R2 of 0.212. Results of the simple and multiple regressions were expressed in crude odds ratios (cOR) and adjusted odds ratios (aOR) with a 95% confidence interval (95%CI), respectively.

### 2.8. Qualitative Study—Design and Analysis

#### 2.8.1. Study Design and Sampling Method

201 eligible Syrian mothers of children aged 0 to 59 months who had previously participated in the quantitative part of the main study and who had given consent to be contacted for the qualitative assessment, were contacted via phone or messenger app. 183 women from four out of six 6 primary health care center study sites were reached and invited to participate in FGD. In total, 43 Syrian women, among those 30 index women and 13 women who were family members (sister, mother or mother-in-law), took part in 11 FGD. 21 Lebanese healthcare staff and technical experts of UNO and NGO were identified through snowball sampling technique and interviewed. Thematic extracts of all FGD and data from four representative KII with healthcare staff (two pediatricians, and two nurses) were sampled for this study.

#### 2.8.2. Qualitative Data Collection

A topic guide for the FGD and a thematic interview guide for the KII on topics related to anemia, complementary feeding diets, and infant and young child feeding practices were developed and approved by the Institutional Review Board of the American University of Beirut prior to the onset of the main study. The FGD were held in spoken Arabic (Northern Levantine dialect) while KII was conducted in English or French. All FGD and KII were recorded after informed consent was sought from participants following a standard ethical protocol. Data saturation was reached after 11 FGD.

#### 2.8.3. Analysis

All FGD and KII were first transcribed from spoken Arabic to phonetic Arabic and then translated into English. All translated FGD and KII were uploaded into the MAXQDA software v.2020 [44]. Qualitative content analysis often referred to as thematic analysis was performed according to the theories of the sociologists Philipp Mayring and Uwe Kuckartz [45,46]. Firstly, relevant sections of the KII or FGD were extracted (referred to as deductive category by Mayring or concept-driven development by Kuckartz) and a coding guideline with main themes was developed (Supplementary Table S2). Secondly, sub-categories were established in an inductive, data-driven fashion (Supplementary Table S3). All steps of the analysis are shown in (Supplementary Figure S2). Reliability tests were performed by two members of the research team to achieve an objective interpretation of results [45].

## 3. Results

### 3.1. Socio-Economic Status

In total, 215 young children aged 6 to 23 months were included in this study. Two in five children were 6 to 11, a third of children were 12 to 17 and a quarter were 18 to 23 months (m) old (Table 1). Half of the mothers were 17 to 25 years of age. About three-quarters of mothers of children aged 6 to 11 months were lactating, while most mothers of children aged 18 to 23 months were non-pregnant and non-lactating. The majority of mothers and fathers had primary or intermediate school education. Yet, secondary school was completed by about one-third of mothers (27.9%), whereas only 7.4% of fathers had completed it. Almost all mothers were housewives with no paid job, whereas the fathers nearly all had a paid job. Most households were registered as refugees and had a monthly income available below 750,000 LBP (equivalent to 500 USD in 2018) for on average 6.3 (±2.9) adults and 2.5 (±1.5) children; 26.3% were classified as food insecure, but only a minority received food assistance (e-voucher) from the World Food Programme (WFP).

### 3.2. Pregnancy Outcomes and Nutritional Status

The majority of children were born full-term through vaginal delivery with a normal birth weight (Table 2). Hence, about a third of the children were considered pre-term, a third were delivered using a Cesarean section and one in five children had a low birth weight. Two in five children were breastfed within the first hour after birth, whereas most children received breastmilk delayed by some hours or even days. Further, the majority of mothers reported to have ever suffered from anemia in the past (67.9%, data not shown) and a quarter of mothers (25.7%) reported to have had anemia during their pregnancy with the child under study. One in five mothers (20.1%) were anemic at the time of data collection, of which most of them (15%) were mildly and a few (5%) were moderately anemic. Two in five children were found to be anemic (42.1%), of which 29.1% were mildly and 13% were moderately anemic. None of the children were severely anemic. The age of the child was neither associated with anemia among children nor with anemia among their mothers (Table 2). The prevalence of acute malnutrition or wasting was 4.2–5.6% according to different classifications applied (WHZ and BAZ in Table 2). Stunting prevalence was at 8.9% for all children, whereas a significantly higher proportion of the children aged 18 to 23 months were suffering from this chronic form of malnutrition (6 to 11 m: 3.4%, 12 to 17 m: 7.1% and 18 to 23 m: 19.3%; *p* < 0.01).

### 3.3. Health Status and Micronutrient Supplementation

The majority of children suffered from any type of symptoms in the past 14 days preceding the study such as fever (48.8%), diarrhea (37.2%), irritability (36.7%), insomnia (27.7%), and a cough or wheeze (27.4%) (Table 3). Most children received medicine and were mostly given pain killers (26.7%) and a few got antibiotics (4.5%). The majority of mothers did not suffer from any type of acute illness (77.3%) or chronic disease (86.9%, data not shown). Yet, the majority of mothers reported symptoms such as fatigue, headache and dizziness (64.3%, 58.2% and 46.5%, respectively).

As far as micronutrient supplementation is concerned, mothers indicated that about half of the 6 to 11 months, a quarter of the 12 to 17 months and a quarter of the 18 to 23 months old children received any type of supplements in the past 6 months (45.3%, 28.6% and 24.1%, respectively; *p* = 0.015). Multivitamins, Vitamin D and iron supplements were mentioned most frequently (31.9%, 30.6% and 25.0%, respectively). Mothers took iron, other single supplements (calcium, magnesium, Vitamin C, Vitamin B1, Vitamin B2) and multivitamins most often (40.0%, 30.9%, 21.8%, respectively), whereas iron-folic acid and Vitamin D were only taken by a few mothers (9.1% and 5.5%, respectively). Nearly all mothers of children aged 12 to 17 months suffered from a symptom of illness and these mothers reported taking significantly more iron supplements as compared to mothers of younger and older children (82.4%, 52.2% and 40.0%, respectively; *p* < 0.05) (Table 3).

### 3.4. Knowledge on Anemia, Dietary Sources of Iron and Main Sources of Health and Nutrition Messages of Mothers of Syrian Refugee Children Aged 6 to 23 Months (Quantitative Assessment)

Nearly all mothers indicated that they heard of anemia before and mentioned most often a lack of iron in the diet (59.2%) as a potential cause for anemia (Table 4). While heavy bleeding was also correctly identified by a few mothers (4.2%), hardly any mother (0.9%) named sickness or infection as a cause of anemia. Dizziness was the most frequently mentioned symptom of anemia followed by paleness, having less energy and blackness under the eyes (57.0%, 27.0%, 22.5% and 11.0%, respectively).

Dark-green vegetables were mentioned most often as a dietary source of iron followed by legumes, the red meat/poultry food group and organ meat (57.2%, 19.2%, 18.3%, 15.4%, respectively). Most mothers stated that they are regularly exposed to health and nutrition messages (69.3%). Healthcare professionals were the main source of these messages followed by all media including conventional and social media, and family and friends (57.2%, 33.5% and 28.4%, respectively). Social media, doctors and the mother’s mother were the most important single source of information while the mother-in-law, community health worker, husband, nurse or midwife, dietitian and pharmacist were mentioned much less often (Table 4).

### 3.5. Dietary Intake of Key Food Groups by Age Group and Anemia Status (24-h DR Data)

Anemic children consumed significantly less dairy, infant formula, (non-Vitamin A-rich) fruits, added fats and oils, zaatar (without oil), animal-source foods as well as iron-rich and iron-fortified foods compared to non-anemic children (Table 5). Further, all anemic children consumed significantly more breastmilk and cow’s milk than their non-anemic peers. In particular, the anemic children aged 12 to 17 months received significantly more cow’s milk, and the children aged 18 to 23 months consumed more breastmilk than their non-anemic counterparts (28.6% vs. 9.8% and 28.6% vs. 5.4%, respectively) (*p* < 0.05).

More age-related differences in the intake of key food groups were found: anemic children aged 6 to 11 months consumed more black tea; anemic children aged 12 to 17 months consumed less added fats and oils and anemic children aged 18 to 23 months consumed significantly fewer fruits than their non-anemic peers (2.2% vs. 14.6%, 85.4% vs. 60.7% and 81.1% vs. 47.6%, respectively) (Table 5).

No significant differences according to age and anemia status were found for the following food groups: grains, roots and tubers, legumes, nuts and seeds, flesh foods, eggs and Vitamin A-rich fruits and vegetables (data not shown).

### 3.6. Determinants of Anemia of Syrian Refugee Children Aged 6 to 23 Months

There were no significant associations neither between anemia and the sex or the age of the child (Table 6). Stunting was not associated with anemia after adjusting for confounders. All other anthropometric outcomes (Table 2) were not associated with anemia (Supplementary Table S4). The Syrian refugee children who were delivered by a Cesarean section had much lower odds of being anemic as compared to children born through vaginal delivery (Table 6). When the child had suffered from any symptom of illness in the past 14 days prior to the survey, he or she was about five times more likely to be anemic. The most common symptom, fever, was not associated with anemia after adjustment in the multivariate analysis (Supplementary Table S4).

Dietary determinants of anemia were identified as follows: children who had not consumed iron-fortified infant formula, added fats and oils or (non-Vitamin A-rich) fruits on the previous day, had two to three times the odds of suffering from anemia (Table 6).

The consumption of breastmilk, cow’s milk (Table 6), dairy, animal-source foods and zaatar (a dried, thyme-sesame powder mix used as a spice) (Supplementary Table S4) the day prior to the survey, were not associated with child anemia after controlling for confounding factors.

Yet, the child had 3.5 times the odds of being anemic if the mother was anemic, too. The child’s intake of any type of supplement in the past 6 months was not associated with child anemia (Table 6). Additionally, if the mother has had anemia in the past, then the child was not more likely to suffer from anemia (Supplementary Table S4).

When the mother did not know that fish or seafood is a dietary source of iron, then the child had nine times the odds to be anemic (Table 6). However, the variable on the mother’s specific knowledge of dizziness as a symptom of anemia was not associated with the child having anemia (Supplementary Table S4).

The variables related to the maternal sources of health and nutrition messages such as social media/internet or nurse/midwife were not associated with child anemia. All socio-economic variables such as the mother’s level of education, the father’s level of education, the number of children and the number of people living in the household, were not associated with the child suffering from anemia. The individual reported food security status of the mother was also not associated with anemia among her child (Supplementary Table S4*).*

### 3.7. Determinants of Moderate and Mild Anemia of Syrian Refugee Children Aged 6 to 23 Months

The following variables were not identified as determinants of moderate or mild anemia: sex and age of the child, type of delivery and the child having had any symptoms in the past 14 days prior to the survey (Table 7).

On the other hand, the timing of breastfeeding initiation after birth was significantly associated with moderate and mild anemia for the category ‘after some hours’ as compared to the category ‘after some days’ showing a protective effect (aOR: 0.18, 95%CI: 0.03; 0.98 and aOR: 0.21, 95%CI: 0.07; 0.68, respectively). Moderate anemia was not associated with the dietary intake of a key food group or single food item after controlling for confounders. Children had about four times the odds of being moderately anemic when they did not receive any type of supplement in the 6 months prior to the study. The odds of being mildly anemic were significantly higher for children who did not consume iron-fortified infant formula (aOR: 2.80, 95%CI: 1.13; 6.91) or added fats and oils (aOR: 2.48, 95%CI: 0.99; 6.20), but lower for children that did not consume cow’s milk (aOR: 0.33, 95%CI: 0.12; 0.92) in the 24 h prior to the study assessment.

Further, the consumption of breastmilk or other fruits was significantly associated with mild anemia in the crude analysis, but not in the adjusted models. For all children whose mother was not currently anemic at the time of data collection, the odds of suffering from moderate or mild anemia were significantly lower (aOR: 0.28, 95%CI: 0.08; 0.96 and aOR: 0.25, 95%CI: 0.10, 0.67). In contrast, the children whose mothers did not know that fish/seafood is a dietary source of iron had about 14 times the odds of suffering from mild anemia as compared to those children whose mothers had this knowledge (Table 7).

### 3.8. Knowledge of, Attitudes towards and Perceptions of Anemia by Syrian Refugee Mothers of Children Aged 0 to 59 Months and Lebanese Primary Healthcare Staff (Qualitative Assessment)

The following paragraphs present findings on the knowledge, attitudes, and perceptions of anemia of Syrian refugee mothers of children aged 0 to 59 months and of selected Lebanese primary healthcare staff. The main themes are summarized in a comparative manner (Table 8) and illustrated by using quotes from the interviewees.

#### 3.8.1. Knowledge of Syrian Refugee Mothers on Anemia

Statements made by mothers reveal that most of them know that anemia and iron deficiency can stem from inadequate nutrition and inappropriate dietary habits. For example, they mentioned that the intake of black tea could “cause deficiencies” (Mother 4) and negatively affect the absorption of iron in the gut (Table 8). Many mothers could name symptoms of anemia by describing the appearance of the children as “very thin and pale” (Mother 2) or behavior such as: “they’re getting dizzy and they’re sitting like this, not participating…” (Mother 2), which can be linked to anemia. Yet, nearly as many mothers were “surprised” (Mother 11) to learn about their child’s anemia diagnosis and described their child’s behavior as “normal” (Mother 13).

#### 3.8.2. Knowledge of Lebanese Healthcare Staff on Anemia

The female Lebanese healthcare staff stated that the main reasons for child anemia among Syrian refugees are malnourishment, a poor diet, lack of knowledge on good nutrition and “many, many convictions [about anemia]”, for instance, the firm conviction that “lentils make anemia stronger” (Doctor 2). The doctors stated that there are inadequate feeding practices such as giving “[black] tea to children who are less than 1 year old” (Doctor 2) or taking not enough “care about the nutrition” (Doctor 1) of each individual child, especially in large families. Further, they also listed financial problems as a cause for malnutrition because parents “can’t afford to buy meat” (Doctor 2) which they deemed a good source of iron. Other causes for anemia were previous illnesses like “pneumonia” (Doctor 1) and stunting among children as a sign of nutritional deficiency and anemia. Symptoms mentioned were “hair loss, fatigue, pallor and asthenia [weakness]” (Doctor 2). (Table 8)

#### 3.8.3. Attitudes of Syrian Refugee Mothers towards Anemia & Potential Treatment Options

The mothers said that they were advised by their doctors to increase the intake of iron-rich foods, such as “chicken and lentils.” (Mother 3). Some women were aware that Vitamin C has a benefitting impact on iron absorption stating that “It [Vitamin C] loosens the blood.” (Mother 9) and that they were told to “eat tomatoes, and […] lemons, too” (Mother 5). However, many mothers “didn’t do anything [after anemia diagnosis of her son]” (Mother 13) and continued their cooking routine: “No, it’s still the same, the food that I cook.” (Mother 14). Yet, many mothers were aware of the negative effect of black tea and “stopped giving him [son with anemia] [black] tea” (Mother 2). Some mothers expressed problems with eliminating black tea from the child’s diet: “I don’t accept giving it [black tea], but my daughter, she drives me crazy, she loves drinking it. She steals it to have it” (Mother 10).

#### 3.8.4. Attitudes of Lebanese Healthcare Staff towards Anemia and Potential Treatment Options

The healthcare staff reported that they run blood tests and then decide on the treatment. If the child has iron deficiency, the doctors prescribe “iron […] for at least two months” (Doctor 1). One doctor mentioned routine anemia prevention among children aged 4 to 6 months using iron or multivitamin supplements (Table 8). The healthcare staff reported the dietary advice they give to mothers of anemic children: “I advise them [Syrian mothers] to not give tea” (Doctor 1). Further, they advised to increase not only iron-rich foods such as meat and lentils, but also the consumption of Vitamin C-rich foods: “Orange juice they can give, yes. For children, 1 year and older” (Doctor 1). Further, healthcare staff stated that older children should be educated on the topic of nutrition and anemia at school. This should prevent “future generations” (Doctor 2) from a high anemia prevalence.

#### 3.8.5. Perceptions of Syrian Refugee Women on the Severity of Anemia

Syrian women downplayed the symptoms of anemia through generalization “Anyway[s] We All Have Iron Deficiency” (Mother 6) or no reaction to the diagnosis: “It’s Fine, he plays, he eats, his food is good [son has anemia]” (Mother 14).

#### 3.8.6. Perceptions of Lebanese Healthcare Staff on the Severity of Anemia

The Lebanese healthcare staff perceived anemia with a varying degrees of severity. The nurses only reported a low incidence of anemia without complications and a rare incidence of severe cases: “[In] our patients […] we don’t find a lot of iron deficiency.” (Nurse 2). On the other hand, the doctors reported that anemia is a severe disease with “too many cases” (Doctor 1) in the Syrian community.

#### 3.8.7. Iron Supplementation as Treatment for Anemia

Micronutrient deficiencies, malnutrition and inadequate diets were the main reasons for the Lebanese health staff to prescribe iron supplements for infants and young children. “All babies who had intrauterine growth retardation and are premature, we have to start iron because they don’t have any storage from fetal life. (…) They [parents] must not buy chips and potato, save money and buy iron.” (Doctor 1). Many mothers reported that they received iron supplements during pregnancy and postpartum. “At the beginning of the pregnancy, I didn’t eat anything; I didn’t have an appetite for food (first 4 to 5 months). After that, my appetite and food intake improved. […] I didn’t add anything, just the iron.” (Mother 18)

The perception of the use of iron pills as treatment option was evaluated with the issue of compliance of the patients to the medical treatment protocol in mind. Some mothers reported that they were not able to follow the protocol because “they (staff) don’t have [iron pills available]” (Mother 12) and were mostly unaware of financial support resulting in “not [getting] it [the prescribed iron pills] for him [son with anemia]” (Mother_14). According to the Lebanese healthcare staff, compliance with the treatment protocol was hampered by several different factors such as the availability of iron pills: “We don’t have iron here [at the primary healthcare center]” (Nurse 2). Additionally, they mentioned that available iron supplements “are primitive […] we don’t have iron [pills] like Fer [heme iron], the strong iron” (Nurse 2). While the healthcare staff were aware that “supplementation for children is not free. Iron is not free” (Doctor 2), they acknowledged the possibility of “large discounts” (Nurse 2).

Yet, some mothers explained that their children or themselves did not accept taking iron supplements. “Yes, it’s the kids it’s [the iron pills] not accepted or easily taken this medication.” (Nurse 1) “The doctor used to give me a pill [iron pill], but I didn’t take it a lot, it was a big pill. I couldn’t swallow it...” (Mother 13). Mothers mentioned that health side effects were the main reason to quit the treatment prematurely. “Yes, but I haven’t taken any [iron pills] for 2 months because my stomach hurts [after taking the pills].” (Mother 8) Lebanese healthcare staff confirmed that the women complained about vomiting as the leading side effect: “But they told us they [the children] don’t take it [the iron pills], because they vomit after it [taking the iron pills].” (Doctor 1). “If the mother comes and wants to feed her child and give him the iron to drink directly after, it’s normal that they’d [the children] vomit.” (Nurse 2). However, the healthcare staff stated that they see health benefits in using the iron pill as a treatment: “Those [iron pills] are for the kids you’re talking about, there’s a lot of improvement using them [iron pills].” (Nurse 2)

Financial constraints were mentioned by the Syrian mothers as an obstacle related to iron pill treatment. “I have my son, the 6-year-old. He’s very thin, when I took him to the doctor, he prescribed vitamins [iron pills], vitamins [iron pills] vary from 13,000 LBP and above, so I don’t get it for him…” (Mother 14). There were also critical statements from the healthcare staff that they knew Syrian mothers could not afford the iron pills, especially the ones of good quality. “So now they […] prescribed the iron supplement that they [the parents] will buy from outside. And maybe this is why they say it’s expensive because it [iron pills from the primary healthcare center] was not effective” (Nurse 1).

## 4. Discussion

### 4.1. Anemia Prevalence and Other Types of Malnutrition among Children

To the best of our knowledge, this is the first study to analyze the dietary and socio-economic determinants of anemia among Syrian refugee children aged 6 to 23 months living in Lebanon under consideration of quantitative and qualitative assessments of the knowledge, attitudes and perceptions of their mothers and the local healthcare staff on the topic of nutritional anemia, its causes as well as its available treatment options at primary care level.

In our main study (*n* = 433), the prevalence of anemia among Syrian refugee children aged 6 to 59 months living in Greater Beirut, Lebanon, was found to be 34% comprising of 24% and 10% of mild and moderate anemia, respectively [18]. The Syrian refugee children aged 6 to 23 months included in this study had a prevalence of anemia of 42% among which 29% and 13% were classified as mild and moderate anemia reflecting a severe public health problem according to the WHO [47]. In 2013, an equally high prevalence of anemia among Syrian refugee children aged 6 to 23 months was found in North Lebanon (43%), but a lower prevalence (28%) among those living in Beirut. Yet, in 2014, anemia among Syrian refugee children of the same age living in the Zaatari refugee camp in Jordan, another host country of Syrian refugees, was reported to be much higher (64%) than in our study. [27,48] A similar study was carried out among children aged 4 to 26 months in Damascus city, Syria, (2011 to 2015) and it was found that 56% of them suffered from iron deficiency anemia. [49] This suggests that iron deficiency is one of the main causes of anemia in our study sample. About a third of the children in our study (29%) suffered from mild anemia and one in six children (13%) suffered from moderate anemia, while severe anemia was not prevalent. Hossain et al. (2016) equally did not find severe anemia among Syrian refugee children in Beirut, but recorded about half of the prevalence figures for mild and moderate anemia in 2013 [27]. Hence, our findings indicate a worsening of the situation of anemia among children between 2013 and 2018. In comparison, global data from the FAO and the WHO showed a prevalence of anemia among young Lebanese children aged 0 to 59 months at 28% in 2009 and 23% in 2019 [6,50].

Putting the anemia prevalence into context, the socio-economic variables assessed were not found to be determinants of anemia among the children aged 6 to 23 months. [51] The reason for this might be the relative homogeneity of the study population. In particular, the mother’s education level was not found to be a determinant of child anemia in contrast to existing evidence [14,15,16,21,52,53]. However, this is in line with findings from our main study on the determinants of anemia among Syrian mothers, previous studies conducted in Lebanon among Lebanese children aged 11 to 75 months of age and Lebanese women of reproductive age whereby the authors could not find any associations between socio-economic variables and the hematological status of the respective study participants [18,52,54,55].

Nearly 1 in 10 children aged 6 to 23 months and 1 in 5 children aged 18 to 23 months were classified as being stunted or chronically malnourished in our study. This is worrisome as chronic malnutrition occurring within the first 1000 days of life impairs the cognitive development of the child [56]. Wasting or acute malnutrition among the Syrian refugee children aged 6 to 23 months in this study reached the 5% threshold which reflects a medium public health concern according to the WHO definition [57]. Wasting is a condition that puts young children at risk of pre-mature death or, when the child survives, impairs the child’s health throughout his or her life [58]. Latest research suggests that there is a relationship between stunting and wasting in that the conditions might occur in the same child at the same time or at different times of the child’s life [59]. On the other hand, overweight/obesity was found to be equally prevalent as wasting among children aged 6 to 23 months. Interestingly, in our main study, anemic mothers of children aged 0 to 59 months had about 14 times the odds of suffering from underweight or overweight/obesity [18]. A study from 2017 carried out in Lebanon among Lebanese hospitalized children aged 6 to 59 months found that malnutrition (underweight) and anemia were significantly associated, but the anemia prevalence was much higher than in our study (72%) [60]. Yet, we did not see any association between any type of malnutrition among the children and child anemia in this study. In particular, stunting and child anemia were not associated unlike in studies that were performed in the same age group in other settings [61,62].

### 4.2. Maternal Anemia in the First 1000 Days of a Child

A quarter of mothers reported having had anemia during pregnancy with the index child. Maternal iron deficiency anemia in the neonatal period can lead to complications during delivery and unfavorable birth outcomes such as premature birth and low birth weight as well as result in low neonatal iron stores that can in turn negatively influence iron balance in later stages of infancy and childhood [17]. Nearly one in five children had a low birth weight, but this was not associated with child anemia in this study, in contrast to a study using datasets from 52 Low- and Lower-Middle-Income countries [63].

Maternal anemia was the unique determinant identified for all categories of total, moderate and mild anemia among children in this study. Kay et al. (2019) also found that the prevalence of total anemia in children and women was strongly correlated among displaced women and children in 121 refugee settings worldwide [64]. This finding is in line with our main study and other studies using meta-analyses or advanced statistical modeling approaches [18,65,66].

### 4.3. Health Status and Nutrient Supplement Use

Most mothers reported high levels of ill-health for their children and themselves. Children that suffered from any symptom in the past 14 days prior to the survey, had higher odds of being anemic. Syrian mothers queried did not know that sickness or infection can likewise be a cause or a symptom of anemia among children. Fever, diarrhea, and irritability were reported most frequently, yet no associations with child anemia were found. In 32 sub-Saharan African countries, higher odds of anemia were found among those children aged 6 to 23 months that had a fever in the two weeks preceding the survey [67]. Irritability and pallor are leading signs of iron deficiency anemia [17]. The prevalence of irritability found in this study (37%) was equal to the one described among the children aged 4 to 26 months suffering from iron deficiency in Syria [50]. While most mothers correctly knew that dizziness, pallor and reduced energy are symptoms of anemia, not all of them could link them to their child’s anemia. In contrast, the healthcare staff correctly assigned these symptoms to anemia in young children. Similar observations were made in other studies whereby physicians noticed pallor as a sign of anemia according to pediatric protocol, but the caregivers did not [68,69,70].

Scott et al. (2014) estimated that 1.8 million yearly deaths in children aged 28 days to five years could be averted by increasing hemoglobin levels by 1 g/dL in these children [71]. The WHO recommends daily iron supplementation for three consecutive months in populations where the prevalence of anemia among children aged 6 to 23 months is at 40% and above, even though there might be other causes for anemia not related to iron intake [72]. In this study, if the child had not taken any nutrient supplement in the past 6 months, he or she had higher odds of suffering from moderate anemia. Oral iron supplementation and fortification of foods with multiple micronutrient powders are effective strategies for improving hemoglobin concentrations and controlling nutritional iron deficiency among infants and toddlers [73,74,75,76,77,78,79,80]. The healthcare staff explained the importance of iron supplementation as a treatment option for anemic children when the nutritional intake was inadequate. However, they complained in part about the low effectiveness of the available iron product in raising hemoglobin levels among children, whereas the Syrian mothers mentioned the high costs as a barrier to obtaining an iron supplement prescription for their anemic child. Economic access to primary health care deteriorated in Lebanon since 2018 with a higher proportion of Syrian households mentioning doctor and treatment fees as main barriers [10]. Some doctors also mentioned iron supplements as a key strategy in the prevention of anemia, particularly for children aged 6 to 11 months. However, the WHO pointed out that iron-repleted children might not benefit from this health intervention and that acceptability might be problematic due to the negative side effects on the gastrointestinal system [72]. In fact, vomiting was mentioned as the main side effect and barrier to iron supplementation and compliance with the treatment protocol by mothers and healthcare staff alike.

### 4.4. The Role of the Complementary Feeding Diet and Iron Intake

The pre-agricultural diet had been estimated to have been high in iron and meeting infant and toddler nutrient requirements [81]. Modern diets often deprive children of iron—the trace element functioning as an oxygen binder in hemoglobin within the red blood cell and serving as an essential nutrient for main metabolic pathways. Estimates suggest that, from the age of 6 to 9 months, a term-born child requires iron from the diet as iron stores wean off [82]. According to the WHO, children should be given nutritious foods with a high iron content from 6 months of age onwards coupled with continued breastfeeding [83,84]. Complementary foods and beverages containing iron have been shown to maintain adequate iron status or prevent deficiency among infants at risk of insufficient iron stores or low intake in the first year of life [85]. In our study, infant formula and follow-on young child formula were frequently given to Syrian refugee children instead of breastmilk. About a third of the children aged 6 to 11 months and about half of the children aged 12 to 23 months consumed it. All children aged 6 to 23 months that were not given iron-rich infant formula on the previous day had higher odds of suffering from anemia as compared to those that received it. A randomized controlled trial was recently conducted in European young children aged 1 to 3 years whereby they found that the probability of iron deficiency after the intervention was lower in the formula group than the cow’s milk product group [86]. Despite its potential to prevent anemia among young children, the relatively high use of infant formula products, which was also noticed in recent studies from Lebanon, is of concern due to its impact on reducing the duration of breastfeeding [87,88]. The reason is the potential societal and actual costs for an economically deprived population [89,90]. Overall, we saw a low prevalence of prolonged breastfeeding and increased use of infant formula beyond 1 year of age. Lebanese researchers argue that there is a discrepancy between breastfeeding policy endorsement and implementation leaving space for breastmilk substitute companies to place their products in the midst of the “Syrian crisis” and thereby violating the code of marketing of breastmilk substitutes [91,92,93].

All children aged 6 to 23 months that did not consume cow’s milk on the previous day had lower odds of suffering from mild anemia. Cow’s milk consumption in infancy, especially the second part of infancy from 6 months to 1 year, has been associated with anemia in numerous studies due to different mechanisms such as that cow’s milk consumption decreases non-heme iron absorption in the gut and can cause intestinal bleeding in the infant [94,95,96,97,98,99,100]. Less than half of the children aged 6 to 17 months consumed non-dairy animal-source foods on the day prior to the survey, hence the children’s diet has more characteristics of a vegetarian diet. However, not consuming a meat product on the day prior to the survey, could not be identified as a determinant of child anemia. Healthcare staff stressed the fact that Syrian refugees face financial constraints which hinder their ability to buy meat products on a regular basis. The most affordable iron-rich foods according to price modeling approaches are dark-green leafy vegetables [101]. Some evidence suggests that vegetarian diets that are varied and nutritionally adequate rarely result in iron and zinc deficiencies [102,103,104]. However, there is also evidence arguing that vegetarian and vegan diets are not safe and can result in micronutrient deficiencies which in turn can affect the brain development of the child [105,106,107]. Children that did not consume fruits on the previous day, had higher odds of suffering from anemia in our study. The doctors stated that they advised mothers to increase the consumption of Vitamin C-rich foods and drinks such as orange juice. Nutritional advice that aims to improve iron status should emphasize not only rich sources of iron but also factors that may enhance or inhibit absorption. Strategies to optimize iron status in this vulnerable age group include consuming Vitamin C-rich fruit or drinks and avoiding tea with meals [108]. The interviewed Lebanese doctors mentioned that they advise Syrian refugee mothers to avoid giving black tea to children, but mothers found it hard to put it into practice due to opposing cultural norms and dietary habits.

### 4.5. Maternal Knowledge on Anemia and the Perspective of Local Healthcare Staff

Our findings showed that the child had much higher odds of suffering from total anemia and mild anemia when the mother did not know that fish/seafood is a good source of dietary iron. In the quantitative assessment of maternal knowledge, most Syrian mothers correctly mentioned the lack of iron in the diet as a cause of anemia. Yet, they listed as the main dietary sources of iron, dark green vegetables before the heme iron sources—meat, organ meat, fish/seafood, followed by the non-heme iron source—legumes, and then a potentially important iron source for young children—iron-fortified breakfast cereals [109,110]. Iron-fortified breakfast cereals were consumed by about a fifth of infants aged 6 to 11 months—lower than in a recent study from North Lebanon, and its consumption was not associated with anemia among the children in our study [111]. Astonishingly, the Lebanese doctors pointed out that some Syrian mothers have the conviction that lentils exacerbate anemia. This highlights the fact that adequate knowledge of a nutritious diet for young children is an important factor in preventing child anemia as demonstrated in other studies globally [23,112]. The key single sources of health and nutrition messages listed by the Syrian mothers were the doctors, their own mothers and social media/internet. There was no association between child anemia and sources of messages in this study. In our main study, the Syrian mothers had lower odds of suffering from anemia themselves, when they heard health and nutrition messages from multiple sources [18]. Yet, in our sub-study of children aged 0 to 5 months, misinformation from the mother-in-law was identified as a barrier to exclusive breastfeeding, which highlights the fact that correct nutritional information needs to be conveyed to do no harm to the child’s nutritional status [113].

By recognizing the multifaceted nature of anemia, mild anemia might be tackled through more intensive nutrition education and counseling approaches emphasizing an iron-rich complementary feeding diet in combination with social media campaigns as has been proven successful elsewhere [114]. For moderate anemia, which requires more context-specific research, the supplementation or fortification of foods or drinks with iron might be the only affordable and feasible treatment option available to this date [115].

### 4.6. Limitations

The results of this study need to be considered in light of the following limitations. The study sample is a convenience sample of the main study conducted in six primary health care centers in Greater Beirut, Lebanon, and therefore, is not generalizable to all Syrian refugee children living in this area as a selection bias might have been introduced by the choice of study sites [18]. The cross-sectional design does not allow to interfere with causality and can suffer from “reverse causality”; therefore, the determinants of anemia identified might not have actually caused anemia in the study population, but remain informative for reaching a better understanding of the outcome measure—the prevalence of child anemia [116]. No venous blood and common blood markers for iron deficiency and iron deficiency anemia were taken and hence the etiology of anemia among Syrian children could not be fully elucidated in this study. The 24-h dietary recall data were taken once for a single child but stretched out over several data collection days (Monday to Saturday), and therefore, the data reflect an average dietary intake of these children aged 6 to 23 months. The five-step, multiple pass 24-h dietary recall method aims to reduce memory lapses, but under- or overreporting might have still occurred [117]. The assessment and interpretation of the qualitative data were carried out with a cultural-sensitive approach, yet elements of subjectivity reflecting the worldview of the enumerators and research team cannot fully be ruled out [118].

## 5. Conclusions

This study highlights the high burden of anemia among children aged 6 to 23 months of a refugee population and its key determinants—the most important one identified was the mother having anemia, too. The role of the quality of complementary feeding diets in the genesis of anemia and likewise its treatment has been largely overlooked. Anemia as a severe public health problem must be more decisively accounted for when designing mother and child health and nutrition programs in countries affected by multiple crises. The effects of the COVID-19 pandemic, the exacerbating financial crisis, the aftermath of the Beirut port explosion in August 2020 whereby the national grain stocks were destroyed, as well as of the Ukrainian crisis impact the food availability and food prices in Lebanon and a worsening of the food security and nutritional status of especially the young children is to be expected calling for immediate emergency interventions [119,120,121,122]. Yet, at the same time, long-term measures such as the implementation of national policies that protect good infant and young child feeding practices need to be facilitated. Mothers should be trained to recognize symptoms of anemia in their young children and consequently be enabled to access adequate treatment and nutritious foods.

Future research should explore the affordability and nutrient adequacy of an improved complementary feeding diet with and without supplementation to put forward a more differentiated approach for children that just start to eat and are most vulnerable to suffer from anemia (6 to 11 months) and children that are able to eat family foods (from 12 months onwards).

## Figures and Tables

**Table 1:** Socio-economic status of Syrian refugee children aged 6 to 23 months.

Socio-Economic Variables ^a^	6–11 m *n* = 87	12–17 m *n* = 70	18–23 m *n* = 58	Total (6–23 m) *n* = 215 ^b^	*p*-Value ^c^
**Child’s sex**					
Male	41 (47.1)	39 (55.7)	29 (50.9)	109 (50.7)	0.564
Female	46 (52.9)	31 (44.3)	28 (49.1)	105 (48.8)	
**Mother’s age (years)**	26.62 ± 5.43	25.45 ± 5.49	27.99 ± 5.67	26.61 ± 5.58	**0.037**
17 to 25 years	39 (44.8)	37 (52.9)	20 (34.5)	96 (44.7)	0.073
25 to 29 years	25 (28.7)	21 (30.0)	15 (25.9)	61 (28.4)	
≥30 years	23 (26.4)	12 (17.1)	23 (39.7)	58 (27.0)	
**Mother’s reproductive status**					
Pregnant	11 (12.6)	16 (22.9)	14 (24.1)	41 (19.1)	**<0.001**
Lactating	63 (72.4)	26 (37.1)	10 (17.2)	99 (46.0)	
Non-pregnant non-lactating	13 (14.9)	28 (40.0)	34 (58.6)	75 (34.9)	
**Mother’s education level**					
No schooling/Illiterate	15 (17.4)	7 (10.1)	8 (13.8)	30 (14.0)	0.712
Primary, Intermediate school	46 (53.5)	43 (63.2)	33 (56.9)	122 (56.7)	
Secondary school and higher	25 (29.1)	18 (26.5)	17 (29.3)	60 (27.9)	
**Father’s education level**					
No schooling/Illiterate	19 (21.8)	10 (14.3)	8 (13.8)	37 (17.2)	
Primary, Intermediate school	65 (74.7)	50 (71.4)	46 (79.3)	161 (74.9)	0.377
Secondary school and higher	3 (3.4)	9 (12.9)	4 (6.9)	16 (7.4)	
**Mother’s employment status**					
No paid job/Housewife	80 (95.2)	68 (97.1)	56 (98.2)	204 (96.7)	0.598
Paid job (daily/part-/full-time)	4 (4.8)	2 (2.9)	1 (1.8)	7 (3.3)	
**Father’s employment status**					
No paid job	4 (4.7)	1 (1.4)	0 (0)	5 (2.3)	0.525
Paid job (daily/part-/full-time)	79 (91.8)	68 (97.1)	57 (98.3)	110 (95.3)	
**Number of children in the household**	2.57 ± 1.41	2.10 ± 1.42	2.79 ± 1.67	2.48 ± 1.51	**0.025**
1 to 2	48 (55.2)	48 (69.6)	33 (56.9)	129 (60.3)	0.156
3+	39 (44.8)	21 (30.4)	25 (43.1)	85 (39.7)	
**Number of people in the household**	6.60 ± 2.74	5.80 ± 3.25	6.26 ± 2.89	6.25 ± 2.96	0.241
1 to 5	33 (38.4)	42 (60.0)	28 (49.1)	103 (48.4)	**0.027**
6 to 20	53 (61.6)	28 (40.0)	29 (50.9)	110 (51.6)	
**Monthly household income**					
≤750,000 LBP (≤500 USD)	57 (69.5)	36 (57.1)	36 (63.2)	129 (63.9)	0.304
>750,000 LBP (>500 USD)	25 (30.5)	27 (42.9)	21 (36.8)	73 (36.1)	
**UNHCR refugee registration status**					
Not registered	14 (16.5)	16 (23.2)	10 (17.9)	40 (19.0)	0.553
Registered	71 (83.5)	53 (76.8)	46 (82.1)	170 (81.0)	
**Receiving WFP food assistance (e-voucher)**					
No	81 (93.1)	69 (98.6)	51 (87.9)	201 (93.5)	**0.043**
Yes	6 (6.9)	1 (1.4)	7 (12.1)	14 (6.5)	
**Food security status**					
Food secure	58 (68.2)	48 (70.6)	42 (73.7)	42 (73.7)	0.784
Food insecure	27 (31.8)	20 (29.4)	15 (26.3)	15 (26.3)	
**Length of stay in Lebanon (months)**	37.80 ± 26.37	37.34 ± 40.69	35.91 ± 29.59	37.14 ± 32.42	0.942

^a^ Categorical variables are expressed in *n* (%) and numerical variables in mean ± SD. ^b^ Lack of corresponding sum of frequencies is due to missing data. ^c^ Significant different at *p*-value < 0.05 (in bold).

**Table 2:** Pregnancy outcomes and nutritional status of Syrian refugee children aged 6 to 23 months and their mothers.

Variables ^a^	6–11 m *n* = 87	12–17 m *n* = 70	18–23 m *n* = 58	Total (6–23 m) *n* = 215 ^b^	*p*-Value ^c^
**Pregnancy outcomes**					
Type of delivery * Vaginal delivery Cesarean section	56 (64.4) 31 (35.6)	44 (62.9) 26 (37.1)	45 (80.4) 11 (19.6)	145 (68.1) 68 (31.9)	0.070
Gestational age of children * Full-term (≥9 months) Pre-term (<9 months)	8.94 ± 0.38 69 (80.2) 17 (19.8)	8.83 ± 0.59 51 (73.9) 18 (26.1)	8.79 ± 0.51 43 (74.1) 15 (25.9)	8.86 ± 0.49 163 (76.5) 50 (32.5)	0.140 0.576
Child’s birth weight * Low birth weight (<2.5 kg) Normal birth weight (2.5–4.2 kg) High birth weight (>4.2 kg)	2.90 ± 0.60 17 (20.7) 64 (78.0) 1 (1.2)	3.00 ± 0.61 8 (12.5) 54 (84.4) 2 (3.1)	2.95 ± 0.78 12 (21.4) 43 (76.8) 1 (1.8)	2.95 ± 0.66 37 (18.3) 161 (79.7) 4 (2.0)	0.688 0.613
Initiation of breastfeeding at birth * Did not breastfeed Immediately within 1 h after birth During the first day after birth (>1 to ≤24 h) <1 to 5 days	3 (3.4) 32 (36.8) 29 (33.3) 23 (26.4)	2 (2.9) 28 (40.6) 25 (36.2) 14 (20.3)	2 (2.9) 24 (41.4) 19 (32.8) 13 (22.4)	7 (3.3) 84 (39.3) 73 (34.1) 50 (23.4)	0.984
Mother had anemia during pregnancy * No Yes	65 (75.6) 21 (24.4)	52 (74.3) 18 (25.7)	42 (72.4) 16 (27.6)	159 (74.3) 55 (25.7)	0.913
**Maternal anemia status ^d^** Not anemic Anemic Mildly anemic Moderately anemic	70 (80.5) 17 (19.5) 14 (16.1) 3 (3.4)	56 (81.2) 13 (18.8) 8 (11.6) 5 (7.2)	45 (77.6) 13 (22.4) 10 (17.2) 3 (5.2)	171 (79.9) 43 (20.1) 32 (15.0) 11 (5.1)	0.870 0.748
**Nutritional status of children**					
Hemoglobin levels (g/dL)	11.09 ± 1.02	11.13 ± 1.17	11.46 ± 1.41	11.21 ± 1.19	0.155
**Child anemia status ^d^**					
Not anemic Anemic Mildly anemic Moderately anemic	46 (52.9) 41 (47.1) 31 (36.0) 10 (11.6)	41 (59.4) 28 (40.6) 18 (26.1) 10 (14.5)	37 (63.8) 21 (36.2) 13 (22.4) 8 (13.8)	124 (57.9) 90 (42.1) 62 (29.1) 28 (13.1)	0.408 0.458
**Weight-for-length/height Z-score (WHZ)**	−0.09 ± 1.13	0.04 ± 1.20	0.22 ± 1.05	0.04 ± 1.13	0.491
Wasting (WHZ < −2)	3 (3.5)	4 (5.7)	2 (3.5)	9 (4.2)	0.762
No wasting (WHZ ≥ −2)	83 (96.5)	66 (94.3)	55 (96.5)	204 (95.8)	
**BMI-for-age Z-score (BAZ)**	−0.04 ± 1.77	0.00 ± 1.24	0.34 ± 1.09	0.08 ± 1.45	0.265
Wasting (BAZ < −2) Normal weight (−2 ≤ BAZ ≤ +2)	5 (5.7) 77 (88.5)	5 (7.1) 62 (88.6)	2 (3.5) 53 (93.0)	12 (5.6) 192 (89.7)	0.873
Overweight/obese (BAZ > +2)	5 (5.7)	3 (4.3)	2 (3.5)	10 (4.7)	
**Weight-for-age Z-score (WAZ)** Underweight (WAZ < −2) No underweight (WAZ ≥ −2)	0.12 ± 1.27 2 (2.3) 85 (97.7)	0.12 ± 1.24 2 (2.9) 68 (97.1)	−0.05 ± 1.26 3 (5.3) 54 (94.7)	0.07 ± 1.26 7 (3.3) 207 (96.7)	0.683 0.603
**Length/Height-for-age Z-score (L/HAZ)**	0.27 ± 1.40	0.25 ± 1.71	−0.34 ± 1.66	0.10 ± 1.60	0.201
Stunting (L/HAZ < −2)	3 (3.4)	5 (7.1)	11 (19.3)	19 (8.9)	**0.004**
No stunting (L/HAZ ≥ −2)	84 (96.6)	65 (92.9)	46 (80.7)	195 (91.1)	

^a^ Categorical variables are expressed in *n* (%) and numerical variables in mean ± SD. ^b^ Lack of corresponding sum of frequencies is due to missing data. ^c^ Significantly different at *p*-value < 0.05 (in bold); ^d^ Anemia status was defined according to WHO hemoglobin cut-off points for women of reproductive age and children aged 6 to 23 months. * Data self-reported by mothers.

**Table 3:** Health status and supplements use of Syrian refugee children aged 6 to 23 months and their mothers.

Variables ^a^	6–11 m *n* = 87	12–17 m *n* = 70	18–23 m *n* = 58	Total (6–23 m) *n* = 215 ^b^	*p*-Value ^c^
**Child had symptom in the past 14 days ^d^** None Yes Fever Diarrhea Irritability Insomnia Cough/wheeze Runny nose/cold Vomiting Fatigue	16 (18.4) 71 (81.6) 42 (48.3) 38 (43.7) 30 (34.5) 18 (20.7) 26 (29.9) 18 (20.7) 16 (18.4) 15 (17.2)	10 (14.3) 60 (85.7) 40 (57.1) 23 (32.9) 28 (40.0) 14 (20.0) 17 (24.3) 16 (22.9) 10 (14.3) 12 (17.1)	11 (19.0) 47 (81.0) 23 (39.7) 19 (32.8) 21 (36.2) 6 (10.3) 16 (27.6) 14 (24.1) 7 (12.1) 5 (8.6)	37 (17.2) 178 (82.8) 105 (48.8) 80 (37.2) 79 (36.7) 38 (27.7) 59 (27.4) 48 (22.3) 33 (15.3) 32 (14.9)	0.730 0.142 0.270 0.772 0.229 0.737 0.880 0.560 0.292
**Mother had an acute illness ^d^** None Yes Flu	61 (70.9) 25 (29.1) 16 (18.6)	56 (81.2) 13 (18.8) 8 (11.6)	46 (82.1) 10 (17.9) 0 (0)	163 (77.3) 48 (22.7) 24 (11.4)	0.200 **0.003**
**Mother had symptom in the past 14 days ^d^** None Yes Fatigue Headache Dizziness Insomnia Loss of appetite Difficulty concentrating Shortness of breath	19 (22.1) 67 (77.9) 47 (54.7) 44 (51.2) 29 (33.7) 23 (26.7) 24 (27.9) 23 (26.7) 14 (16.3)	2 (2.9) 68 (97.1) 56 (80.0) 47 (67.1) 45 (64.3) 26 (37.1) 28 (40.0) 31 (44.3) 27 (38.6)	6 (10.5) 51 (89.5) 34 (59.6) 33 (57.9) 25 (43.9) 23 (40.4) 20 (35.1) 12 (21.1) 18 (31.6)	27 (12.7) 186 (87.3) 137 (64.3) 124 (58.2) 99 (46.5) 72 (33.8) 72 (33.8) 66 (31.0) 59 (27.7)	**0.001** **0.003** 0.132 **0.001** 0.187 0.275 **0.010** **0.006**
**Child took medicine in the past 14 days ^d^** None Yes Pain killers	33 (41.8) 46 (58.2) 25 (31.6)	29 (43.3) 38 (56.7) 18 (26.9)	18 (32.1) 38 (67.9) 11 (19.6)	80 (39.6) 122 (60.4) 54 (26.7)	0.399 0.299
**Mother currently takes medicine ^d^** None Yes ^e^	77 (88.5) 10 (11.5)	62 (88.6) 8 (11.4)	42 (72.4) 16 (27.6)	181 (84.2) 34 (15.8)	**0.016**
**Child took supplements in the past 6 months ^d^** None Yes Multivitamins Vitamin D Iron	47 (54.7) 39 (45.3) 10 (26.3) 14 (36.8) 8 (21.1)	50 (71.4) 20 (28.6) 9 (45.0) 5 (25.0) 3 (15.0)	44 (75.9) 14 (24.1) 4 (28.6) 3 (21.4) 7 (50.0)	141 (65.9) 73 (34.1) 23 (31.9) 22 (30.6) 18 (25.0)	**0.015** 0.334 0.461 **0.049**
**Mother takes any type of supplement ^d^** None Yes Iron Multivitamins Iron-Folic Acid	62 (72.1) 24 (27.9) 12 (52.2) 4 (17.4) 1 (4.3)	53 (75.7) 17 (24.3) 14 (82.4) 4 (23.5) 1 (5.9)	43 (74.1) 15 (25.9) 6 (40.0) 4 (26.7) 3 (20.0)	158 (73.8) 56 (26.2) 32 (58.2) 12 (21.8) 5 (9.1)	0.887 **0.040** 0.775 0.307

^a^ Categorical variables are expressed in *n* (%) and numerical variables in mean ± SD. ^b^ Lack of corresponding sum of frequencies is due to missing data. ^c^ Significantly different at *p*-value < 0.05 (in bold); ^d^ Multiple response question. ^e^ Type of medicine taken against allergies, for asthma and thyroid medication, oral contraceptive.

**Table 4:** Knowledge of mothers of Syrian refugee children aged 6 to 23 months on anemia and sources of health and nutrition messages (Quantitative assessment, Source: Questionnaire).

Variables ^a^	6–11 m *n* = 87	12–17 m *n* = 70	18–23 m *n* = 58	Total (6–23 m) *n* = 215 ^b^	*p*-Value ^c^
**Maternal knowledge**					
Heard of anemia before No Yes	6 (6.9) 81 (93.1)	3 (4.3) 67 (95.7)	4 (6.9) 54 (93.1)	13 (6.0) 202 (94.0)	0.818
**Causes of anemia ^d^** Lack of iron in the diet Heavy bleeding (menstruation) Sickness/infection	55 (63.2) 1 (1.1) 2 (2.3)	39 (55.7) 2 (2.9) 0 (0)	32 (57.1) 6 (10.7) 0 (0)	126 (59.2) 9 (4.2) 2 (0.9)	0.597 **0.023** 0.341
**Symptoms of anemia ^d^** Dizziness Paleness Less energy Black under the eyes Spoon/Bent nails More likely to become sick	38 (47.5) 23 (28.7) 15 (18.8) 11 (13.8) 2 (2.5) 1 (1.3)	40 (60.6) 19 (28.8) 14 (21.1) 4 (6.1) 1 (1.5) 0 (0)	36 (66.7) 12 (22.2) 16 (29.6) 7 (13.0) 1 (1.9) 0 (0)	114 (57.0) 54 (27.0) 45 (22.5) 22 (11.0) 4 (2.0) 1 (0.5)	0.073 1.000 0.318 0.292 1.000 1.000
**Dietary sources of iron ^d^** Dark-green vegetables Legumes Red meat/poultry Organ meat Iron-fortified breakfast cereal Fish/seafood	46 (54.8) 16 (19.0) 17 (20.2) 12 (14.3) 11 (13.1) 8 (9.5)	43 (63.2) 11 (16.2) 10 (14.7) 12 (17.6) 4 (5.9) 5 (7.4)	30 (53.6) 13 (23.2) 11 (19.6) 8 (14.3) 10 (17.9) 3 (5.4)	119 (57.2) 40 (19.2) 38 (18.3) 32 (15.4) 25 (12.0) 16 (7.7)	0.468 0.612 0.648 0.820 0.115 0.681
**Sources of health and nutrition messages**					
Hears messages No Yes	29 (33.3) 58 (66.7)	19 (27.1) 51 (72.9)	18 (31.0) 40 (69.0)	66 (30.7) 149 (69.3)	0.704
Healthcare Professionals ^d^ Doctor Community Health Worker Nurse/midwife Dietitian/Nutrition education class Pharmacist	50 (57.5) 17 (19.5) 4 (4.6) 3 (3.4) 0 (0) 1 (1.1)	43 (61.4) 15 (21.4) 6 (8.6) 4 (5.7) 2 (2.9) 1 (1.4)	30 (51.7) 7 (12.1) 0 (0) 0 (0) 5 (8.6) 0 (0)	123 (57.2) 39 (18.1) 10 (4.7) 7 (3.3) 7 (3.3) 2 (0.9)	0.542 0.356 0.059 0.205 **0.008** 1.000
Family and friends ^d^ Mother Friends/neighbors Mother-in-law Husband	22 (25.3) 11 (12.6) 5 (5.7) 6 (6.9) 3 (3.4)	18 (25.7) 17 (24.3) 3 (4.3) 2 (2.9) 1 (1.2)	21 (36.2) 11 (19.0) 10 (17.2) 4 (6.9) 4 (6.9)	61 (28.4) 39 (18.1) 18 (8.4) 12 (5.6) 8 (3.7)	0.301 0.167 **0.026** 0.491 0.303
All media ^d^ Social media/Internet Media (Radio/TV)	27 (31.0) 19 (21.8) 12 (13.8)	22 (31.4) 15 (21.4) 11 (15.7)	23 (39.7) 14 (24.1) 13 (22.4)	72 (33.5) 48 (22.3) 36 (16.7)	0.507 0.926 0.380

^a^ Categorical variables are expressed in *n* (%). ^b^ Lack of corresponding sum of frequencies with total sample size is due to missing data. ^c^ Significantly different at *p*-value < 0.05 (in bold); X^2^—analysis and Fisher’s exact test were used for categorical variables. ^d^ Multiple response question, sums of frequencies do not add up.

**Table 5:** Dietary intake of key foods and food groups of Syrian refugee children aged 6 to 23 months according to age and anemia status (24-h DR data).

Variables ^a^	6–11 m *n* = 87		12–17 m *n* = 70		18–23 m *n* = 58		Total (6–23 m) *n* = 214 ^b^	
Key Foods & Food Groups	Not Anemic (*n* = 46)	Anemic (*n* = 41)	Not Anemic (*n* = 41)	Anemic (*n* = 28)	Not Anemic (*n* = 37)	Anemic (*n* = 21)	Not Anemic (*n* = 124)	Anemic (*n* = 90)
Breastmilk	32 (69.6)	33 (80.5)	17 (41.5)	12 (42.9)	**2 (5.4)**	**6 (28.6)**	**51 (41.1)**	**51 (56.7)**
Dairy Infant formula Yogurt Cheese Cow’s milk	**35 (76.1)** 21 (45.7) 19 (41.3) 7 (15.2) 9 (19.6)	**23 (56.1)** 11 (26.8) 14 (34.1) 4 (9.8) 9 (22.0)	36 (87.8) 24 (58.5) 23 (56.1) 6 (14.6) **4 (9.8)**	24 (85.7) 13 (46.4) 13 (46.4) 5 (17.9) **8 (28.6)**	35 (94.6) 25 (67.6) 18 (48.6) 15 (40.5) 5 (13.5)	17 (81.0) 9 (42.9) 8 (38.1) 5 (23.8) 6 (28.6)	**106 (85.5)** **70 (56.5)** 60 (48.4) 28 (22.6) **18 (14.5)**	**64 (71.1)** **33 (36.7)** 35 (38.9) 14 (15.6) **23 (25.6)**
Other fruits & vegetables Other fruits Other vegetables	21 (45.7) 20 (43.5) 8 (17.4)	18 (43.9) 10 (24.4) 13 (31.7)	27 (65.9) 18 (43.9) 20 (48.8)	18 (64.3) 15 (53.6) 10 (35.7)	5 (13.5) **30 (81.1)** 18 (48.6)	4 (19.0) **10 (47.6)** 11 (52.4)	80 (64.5) **68 (54.8)** 46 (37.1)	53 (58.9) **35 (38.9)** 34 (37.8)
Added fats & oils ^c^	26 (56.5)	20 (48.8)	**35 (85.4)**	**17 (60.7)**	36 (97.3)	20 (95.2)	**97 (78.2)**	**57 (63.3)**
Animal-source foods (ASF) Non-Dairy ASF	38 (82.6) 11 (23.9)	27 (65.9) 10 (24.4)	38 (92.7) 19 (46.3)	25 (89.3) 11 (39.3)	36 (97.3) 22 (59.5)	20 (95.2) 15 (71.4)	**112 (90.3)** 52 (41.9)	**72 (80.0)** 36 (40.0)
Iron-rich/-fortified foods Iron-rich foods Iron-fortified cereals	28 (60.9) 8 (17.4) 8 (17.4)	17 (41.5) 8 (19.5) 8 (19.5)	30 (73.2) 14 (34.1) 2 (4.9)	19 (67.9) 10 (35.7) 2 (7.1)	31 (83.8) 21 (56.8) 0 (0)	13 (61.9) 10 (47.6) 0 (0)	**89 (71.8)** 43 (34.7) 10 (8.1)	**90 (42.1)** 28 (31.1) 10 (11.1)
Condiments Zaatar ^d^ (without oil)	24 (52.2) 0 (0)	19 (46.3) 0 (0)	29 (70.7) 7 (17.1)	18 (64.3) 1 (3.6)	31 (83.8) 9 (24.3)	17 (81.0) 3 (14.3)	84 (67.7) **16 (12.9)**	54 (60.0) **4 (4.4)**
Black tea	**1 (2.2)**	**6 (14.6)**	7 (17.1)	3 (10.7)	12 (32.4)	11 (52.4)	20 (16.1)	20 (22.2)

^a^ Categorical variables are expressed in *n* (%). ^b^ Lack of corresponding sum of frequencies with total sample size is due to missing data. Significantly different at *p*-value < 0.05 (in bold). ^c^ includes Zaatar with oil. ^d^ Zaatar: a dried, thyme-sesame powder mix used as a spice.

**Table 6:** Determinants of anemia of Syrian refugee children aged 6 to 23 months.

Child Variables ^a^ (*n* = 214)	Not anemic	Anemic	Child Anemia	
	(*n* = 124)	(*n* = 90)	cOR (95%CI)	aOR (95%CI)
**Sex** Male Female	60 (48.4) 64 (51.6)	49 (55.1) 40 (44.9)	1.31 (0.76; 2.26) 1	1.46 (0.63; 3.37) 1
**Child’s age (months)** 6 to 11 12 to 17 18 to 23	46 (37.1) 41 (33.1) 37 (29.8)	41 (45.6) 28 (31.1) 21 (23.3)	1.57 (0.80; 3.10) 1.20 (0.59; 2.47) 1	1.27 (0.34; 4.72) 1.91 (0.59; 6.19) 1
**Type of delivery** Vaginal delivery Cesarean section	16 (13.0) 70 (56.9)	14 (15.9) 52 (59.1)	1 0.66 (0.37; 1.20)	1 0.30 (0.11; 0.83) *
**Health status**				
**Suffered from any symptoms past 14 days** No Yes	25 (20.2) 99 (79.8)	11 (12.2) 79 (87.8)	1 1.81 (0.84; 3.91)	1 4.98 (1.17; 21.13) *
**Dietary intake (24-h DR)**				
**Breastmilk** No Yes	73 (58.9) 51 (41.1)	39 (43.3) 51 (56.7)	1 1.87 (1.08; 3.24) *	1 0.67 (0.21; 2.09)
**Infant formula** No Yes	54 (43.5) 70 (56.5)	57 (63.3) 33 (36.7)	2.24 (1.28; 3.91) ** 1	3.16 (1.18; 8.42) * 1
**Cow’s milk** No Yes	106 (85.5) 18 (14.5)	67 (74.4) 23 (25.6)	0.50 (0.25; 0.99) * 1	0.43 (0.14; 1.34) 1
**Other fruits** No Yes	56 (45.2) 68 (54.8)	55 (61.1) 35 (38.9)	1.91 (1.10; 3.31) * 1	2.62 (1.07; 6.41) * 1
**Added fats and oils** No Yes	27 (21.8) 97 (78.2)	86 (95.6) 4 (4.4)	2.08 (1.14; 3.80) * 1	3.07 (1.13; 8.30) * 1
**Supplement use in the past 6 months**				
**Taken any supplements** No Yes	74 (60.2) 49 (39.8)	66 (73.3) 24 (26.7)	1 0.55 (0.30; 0.99) *	1 0.43 (0.18; 1.05)
**Maternal Variables**				
**Maternal anemia** Not anemic Anemic	104 (84.6) 19 (15.4)	66 (73.3) 24 (26.7)	1 1.99 (1.01; 3.91) *	1 3.50 (1.15; 10.65) *
**Knowledge on fish/seafood** **as dietary source of iron** No Yes	107 (88.4) 19 (15.4)	84 (97.7) 2 (2.3)	5.50 (1.22; 24.85) * 1	9.26 (1.22; 70.14) * 1

* *p* < 0.05. ** *p* < 0.001. ^a^ Categorical variables are expressed in *n* (%). Lack of corresponding sum of frequencies with total sample size is due to missing data. Model 1: Sex of the child, gestational age, age of the child (months), child suffered from any symptoms the past 14 days, suffered from fever, HAZ, education of father, number of people, number of children, hears about health and nutrition messages: from social media/internet and nurse/midwife, type of delivery, breastmilk, infant formula, other fruits, fish/seafood as source of iron, mothers with anemia: Hb < 12.0 g/dL for non-pregnant and <11.0 g/dL, mother suffered from anemia in the past, dizziness (cause for anemia), cow’s milk, added fats and oils, zaatar, snacks of mother, FIES, timing of breastfeeding initiation, child taken any nutrient supplements in the past 6 months.

**Table 7:** Determinants of moderate and mild anemia of Syrian refugee children aged 6 to 23 months.

Variables	cOR (95%CI) Reference Group: Not Anemic		aOR (95%CI) Reference Group: Not Anemic	
	Moderately Anemic (*n* = 28)	Mildly Anemic (*n* = 62)	Moderately Anemic (*n* = 28)	Mildly Anemic (*n* = 62)
**Child’s sex** Male Female	1.95 (0.83; 4.57) 1	1.12 (0.61; 2.07) 1	1.77 (0.60; 5.23) 1	1.20 (0.55; 2.64) 1
**Child’s age (months)** 6 to 11 12 to 17 18 to 23	1.03 (0.37; 2.87) 1.13 (0.40; 3.16) 1	1.96 (0.90; 4.28) 1.25 (0.54; 2.90) 1	0.57 (0.12; 2.69) 1.06 (0.27; 4.19) 1	1.53 (0.46; 5.14) 1.50 (0.50; 4.50) 1
**Type of delivery** Vaginal delivery Cesarean Section	1.97 (0.74; 5.26) 1	1.38 (0.71; 2.67) 1	1.73 (0.46; 6.57) 1	2.11 (0.85; 5.21) 1
**Breastfeeding initiation** Did not breastfeed Within 1 h after birth After some hours After some days	3.11 (0.42; 22.87) 1.71 (0.59; 4.94) 0.47 (0.13; 1.67) 1	1.33 (0.20; 8.92) 1.37 (0.61; 3.04) 0.72 (0.31; 1.66) 1	1.57 (0.11; 23.36) 0.83 (0.16; 4.18) 0.18 (0.03; 0.98) * 1	0.37 (0.04; 3.74) 0.57 (0.19; 1.75) 0.21 (0.07; 0.68) ** 1
**Dietary intake (24-h DR)**				
**Breastmilk** No Yes	0.59 (0.26; 1.36) 1	0.50 (0.27; 0.92) * 1	0.46 (0.11; 1.95) 1	0.97 (0.33; 2.82) 1
**Infant formula** No Yes	1.52 (0.67; 3.47) 1	2.77 (1.46; 5.27) ** 1	1.18 (0.35; 3.97) 1	2.80 (1.13; 6.91) * 1
**Cow’s milk** No Yes	0.63 (0.22; 1.76) 1	0.45 (0.22; 0.96) * 1	0.41 (0.1; 1.69) 1	0.33 (0.12; 0.92) * 1
**Dietary Intake (24-h DR)** **Other fruits**				
No Yes	1.20 (0.53; 2.72) 1	2.34 (1.24; 4.41) ** 1	1.06 (0.35; 3.26) 1	2.22 (0.97; 5.09) 1
**Added fats and oils** No Yes	2.07 (0.86; 5.03) 1	2.20 (1.12; 4.31) * 1	2.54 (0.71; 9.02) 1	2.48 (0.99; 6.20) * 1
**Supplement use in the past 6 months**				
**Taken any supplements** No Yes	2.98 (1.06; 8.38) * 1	1.47 (0.77; 2.82) 1	3.80 (1.03; 14.09) * 1	1.75 (0.75; 4.10) 1
**Maternal variables**				
**Maternal anemia** Not anemic Anemic	0.55 (0.21; 1.48) 1	0.49 (0.23; 1.03) 1	0.28 (0.08; 0.96) * 1	0.25 (0.10; 0.67) ** 1
**Knowledge on fish/seafood** **as dietary source of iron** No Yes	3.43 (0.43; 27.31) 1	7.66 (0.98; 59.74) 1	7.72 (0.64; 92,70) 1	13.78 (1.38; 137.62) * 1

Model 1: Sex of the child, gestational age, age of the child (months), child suffered from any symptoms the past 14 days, number of people in the household, mother heard health and nutrition messages from social media/internet, type of delivery, breastmilk, infant formula, cow’s milk, non-Vitamin A-rich fruits, added fats/oils, maternal knowledge on fish/seafood as a source of iron, mothers with anemia: Hb < 12.0 g/dL for non-pregnant and <11.0 g/dL, timing of breastfeeding initiation, child taken any nutrient supplements in the past 6 months, FIES. * *p* < 0.05, ** *p* < 0.01.

**Table 8:** Knowledge, attitudes and perceptions on anemia and its treatment options of mothers of Syrian refugee children aged 0 to 59 months (*n* = 43) and Lebanese primary healthcare staff (*n* = 4). (Qualitative Assessment, Source: 11 FGD and 4 KII).

Main Themes (*n* = 7)	Sub-Themes (*n* = 23)	Syrian Refugee Mothers	Lebanese Healthcare Staff
**Knowledge** (*N* = total number of quotes)	(*n* = number of quotes)	*Supportive quotes*	*Supportive quotes*
Causes for anemia (*N* = 362)	Inadequate nutrition/diet (*n* = 57)	*“And the nutrition isn’t complete I mean…” (Mother 1)*	*“The common reason [for anemia] is diet. Most common [reason] is diet.” (Doctor 1)*
	Poor eating Habits (*n* = 244)	*“I eat, average. But my children, they don’t like tomatoes, they don’t like vegetables.” (Mother 2)*	*“The 1st [reason] is that we [Lebanese and Syrians] have bad habits. We take a lot of [black] tea.* *We have a cultural attitude that(...) we give tea even to children who are less than 1 year old. (...)* *The tea, it reinforces anemia.” (Doctor 2)*
	Ignorance (*n* = 11)	*“She doesn’t know where anemia* *comes from.” (Mother 2)*	*“Yes, they [Syrian parents] have the convictions that lentils make anemia stronger (...), spinach enhances anemia.” (Doctor 2)*
	Previous illness (*n* = 12)	--	*“Many [women] have anemia from pneumonia, they don’t eat well after [illness].“ (Doctor 1)*
	Financial constraints (*n* = 38)	--	*“And 4th [reason], the financial means. They can’t afford to buy (...) meat. And they don’t eat it [meat].” (Doctor 2)*
Symptoms of anemia (*N* = 79)	Dizziness (*n* = 40)	*“They [the children] have anemia, you see they’re very thin and pale, and they’re getting dizzy, and they’re sitting like this, not participating…” (Mother 2)*	--
	Fatigue (*n* = 23)	*“After that my body started getting tired. Before I didn’t feel the symptoms. I was energetic, and very well.” (Mother 1)*	*“(…) They don’t have the strength to do anything but sit down.” (Doctor 2)*
	Pallor (*n* = 10)	*“His [son] face was very pale” (Mother 2)*	*“The manifestations are […] hair [loss], fatigue, pallor, asthenia.” (Doctor 2)*
	No recognition of symptoms (*n* = 6)	*“No, no everything was normal [behavior of son after anemia diagnosis].”* *(Mother 13)*	--
Reasons for iron supplementation (*N* = 64)	Inadequate nutrition/diet (*n* = 45)	*“Her [daughter] food intake was low. But after I started giving her iron, her eating habits started to improve.”* *(Mother 18)*	*“They [parents] must not buy chips and potato, save money and buy iron.” (Doctor 1)*
	Pregnancy (*n* = 19)	*“When I was pregnant, I used to take [iron pills]” (Mother 7)*	*“(…) during pregnancy they should take the iron. Because we notice that so many said they had anemia during pregnancy.” (Doctor 1)*
**Attitude**			
Medical approach (*N* = 64)	Blood tests (*n* = 23)	*“He [doctor] told me to do a blood test so I did and from there he told me that the baby has anemia” (Mother 16)*	“With blood test[s] […] it shows hemoglobin, hematocrit and MCV *. So, if I find any abnormal finding, I run more blood tests. *For iron, for thalassemia.” (Doctor 1)*
	Prophylaxis (*n* = 4)	**--**	“We start the prevention for anemia between the ages of four and six months for every baby. It’s a routine recommendation. […] we have to start just the prophylaxis [iron supplements] for anemia in the healthy baby […], if we have a premature baby or any IUGR **, I start it before.” (Doctor 1)
	Iron prescription (*n* = 37)	*“Yes, a few months ago. They gave* *her [daughter] iron.” (Mother 18)*	*“Iron treatment I know, [I prescribe it] at least two months. And I follow up with blood tests.” (Doctor 1)*
Dietary intervention (*N* = 48)	Decreased consumption of iron inhibitors (*n* = 6)	*“They told me he’s anemic, so I stopped giving him [son] tea” (Mother 2)*	*“Every time one [child] come in also I give iron supplementation, I advise them [Syrian mothers] “do not give tea”.” (Doctor 1)*
	Increased consumption of iron-rich foods (*n* = 31)	*“(…) the doctor told her to eat chicken and lentils.” (Mother 13)*	*“Keep up this discipline in food. […] for example, your son likes meat, so yes cook him some meat with soup, with zucchini, with vegetables, […] whatever vegetable is found and has a cheap price; the minimum amount taken out of the salary, the things you’re able to buy.” (Nurse 2)*
	Increased consumption of iron enhancers (*n* = 11)	*“(…) they told me, eat tomatoes, and* *they eat lemons, too.” (Mother 5)*	*“Orange juice they can give, yes. For children, one year and up.” (Doctor 1)*
**Perceptions**			
Degree of severity of anemia (*N* = 50)	Anemia is a severe problem (*n* = 35)	*“They look at her and you get disgusted,* *I mean, (…) from anemia and poor nutrition, they call me from school and tell me to come pick her up. I mean there is no* *solution, the color on her face is scary.” (Mother 10)*	*“I do not have numbers, but I know it [anemia] is* *frequent. But I do not have an exact number of the kids that have anemia.“ (Doctor 2)*
	Anemia is not a severe problem (*n* = 15)	*“Anyways, we all have iron deficiency.” (Mother 6)*	*“But […] in our patients, […] we don’t find a lot* *of iron deficiency.“ (Nurse 2)*
Compliance to iron supplements (*N* = 107)	Availability (*n* = 10)	*“I asked them once and they told me they don’t have [iron pills] available at the PHCC* ^†^*.” (Mother 12)*	*“We don’t have iron here [at the PHCC ^†^].” (Nurse 2)*
	Acceptability (*n* = 30)	*“The doctor used to give me a pill [iron pill], but I didn’t take [it] a lot, it was a big pill. I couldn’t swallow it.* *I used it, but not a lot, but it helped my blood levels improve.” (Mother 13)*	*“Yes, it’s the kids it’s [the iron pills] not accepted [by them] or easily taken [by them], this medication.” (Nurse 1)*
	Health impact (*n* = 35)	*“He [son with anemia] vomits it* *[iron pills]” (Mother 17)*	*“Those [iron pills] are for the kids you’re talking about, there’s a lot of improvement using them [iron pills].” (Nurse 2)*
	Financing (*n* = 32)	*“We don’t have [enough money],* *I’m telling you my husband doesn’t work, we don’t have money* *[to buy iron pills].” (Mother 2)*	*“All medications are provided by the Ministry* *of Public Health, it’s free 100%.” (Nurse 2)*

* MCV: mean corpuscular volume, ** IUGR: intra-uterine growth retardation, ^†^ PHCC: primary health care center.

## Data Availability

The quantitative and qualitative datasets can be made available upon reasonable request to the corresponding author.

## Supplementary Materials

The following supporting information can be downloaded at: https://www.mdpi.com/article/10.3390/nu15030700/s1, Table S1: Definitions of key foods and food groups consumed by Syrian refugee children aged 6 to 23 months (24 h DR data). Figure S1: Analytical framework for the determinants of child anemia; Figure S2: Overview of steps of the qualitative data analysis; Table S2: Coding guidelines of main themes; Table S3: Coding guidelines of sub-themes; Table S4: Additional variables tested as determinants of child anemia.
