# Supplementary material for: Anemia among Syrian Refugee Children Aged 6 to 23 Months Living in Greater Beirut, Lebanon, including the Voices of Mothers’ and Local Healthcare Staff: A Mixed-Methods Study
Source: Nutrients. 2023 Jan 30;15(3):700. doi: 10.3390/nu15030700 (PMC9920708; doi:10.3390/nu15030700)
Supplement: Supplementary file 1 [file nutrients-15-00700-s001.zip › nutrients-2108059-supplementary.pdf]

nutrients MDPI

Supplementary materials

Table S1. Definitions of key foods and food groups consumed by Syrian refugee children aged 6 to 23 months (24h DR data).

Key foods & food groups	Definitions
Breastmilk	Breastmilk
Dairy	Cow's milk (fresh and powder), yoghurt incl. "Ayran" (yoghurt drink) and "keshk" & "kishk" (a dried yoghurt mass); Other dairy products: "akkawi" & "baladi" cheese (soft, unripe cheeses), cheese spreads ("picon" & "smeds"), feta, gruyère cheese, halloumi, "kashkawen" (semi-hard yellow cheese), "labneh" (Lebanese cream cheese), mozzarella and parmesan; infant formula (including low, medium and high iron IF)
Infant formula	Infant formula (incl. low, medium and high iron)
Yogurt	Yoghurt incl. "Ayran" (yoghurt drink) and "keshk" & "kishk"(a dried yoghurt mass)
Cheese	"akkawi" & "baladi" cheese (soft, unripe cheeses), cheese spreads ("picon" & "smeds"), feta, gruyère cheese, halloumi, "kashkawen" (semi-hard yellow cheese), "labneh" (Lebanese cream cheese), mozzarella and parmesan
Cow's milk	Cow's milk (fresh and powder)
Other fruits and vegetables	Other fruits (all non-Vitamin A-rich fruits such as apples, bananas, cherries, dates, grapes, olives, oranges, tangerines, peaches, pears, pineapples, plums, watermelon and pure fruit juices & purées) Other vegetables (all non-Vitamin-A rich vegetables such as cabbage, cauliflower, celery, cucumber, eggplant, green pepper, lettuce, "loubieh" (green beans), mushrooms, okra, radishes, squash and tomatoes)
Other fruits	Other fruits (all non-Vitamin A-rich fruits such as apples, bananas, cherries, dates, grapes, olives, oranges, tangerines, peaches, pears, pineapples, plums, watermelon and pure fruit juices & purées)
Other vegetables	Other vegetables (all non-Vitamin-A rich vegetables such as cabbage, cauliflower, celery, cucumber, eggplant, green pepper, lettuce, "loubieh" (green beans), mushrooms, okra, radishes, squash and tomatoes)
Added fats and oils	All added oils and fats (butter, ghee, corn and olive oil, mutton tallow), "zeit ou zaatar" (a spice mixture based on wild thyme and toasted sesame seeds mixed with olive oil)
Animal-source foods (ASF)	Percentage of children aged 6–23 months who consumed at least one food from the food groups: "Dairy", "Flesh foods" ["Meats" (chicken, beef, lamb and processed products such as ham), "Organ meats" (chicken liver), "Fish/Seafood"] and/or "Eggs"
Non-Dairy ASF	Percentage of children aged 6–23 months who consumed at least one food from the food groups: "Flesh foods" ["Meats" (chicken, beef, lamb and processed products such as ham), "Organ meats" (chicken liver), "Fish/Seafood"] and/or "Eggs"
Iron-rich/-fortified foods	Iron-rich food, iron-fortified cereals, infant formula (iron-rich)

Iron-rich foods	Percentage of children aged 6 to 23 months who consumed at least one food from the food groups: "Legumes" [chickpeas (including "hummus"- a chickpea spread), fava beans, kidney beans, lentils, lima beans and peas)] or "Flesh foods"
Iron-fortified cereals	Iron-fortified breakfast cereals suitable for children aged 6 to 23 months
Condiments	"Zaatar" with and without oil; Other condiments [items commonly used in small quantities and mainly used to enhance the flavor of the dish such as chilies, herbs, ketchup, mayonnaise, mustard, orange blossom water, rose syrup, spices, stocks, vinegar, as well as vegetables such as onions and garlic, cornstarch, leavening agents, sugar, honey and molasses (thick syrup) added to savory dishes]
Zaatar (without oil)	"Zaatar" without oil – a dried, thyme and sesame powder mix used as a spice
Black tea	Black tea (common brand used: Lipton tea)

Figure S1. Analytical framework for the determinants of child anemia

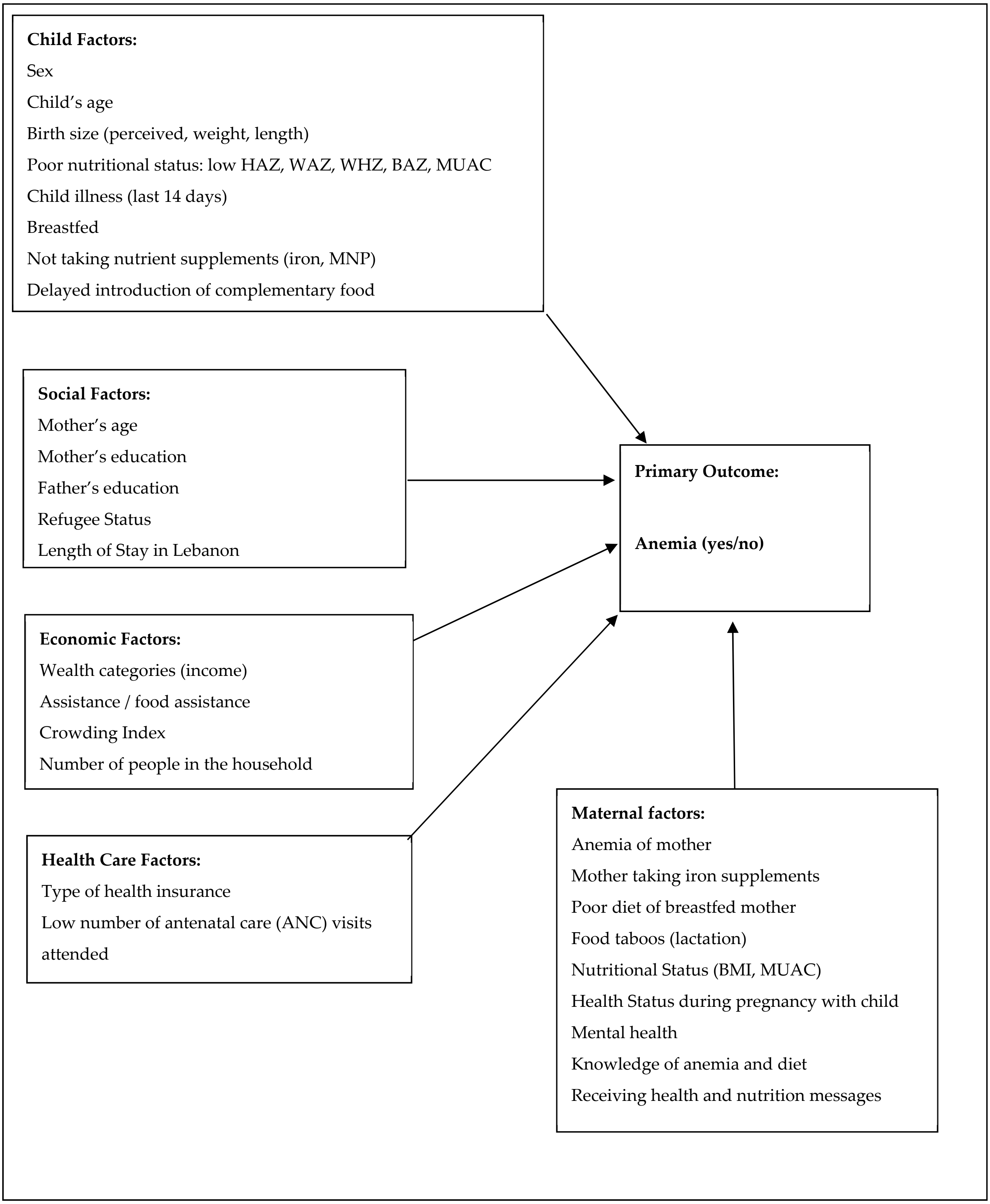

Figure S2. Overview of steps of the qualitative data analysis

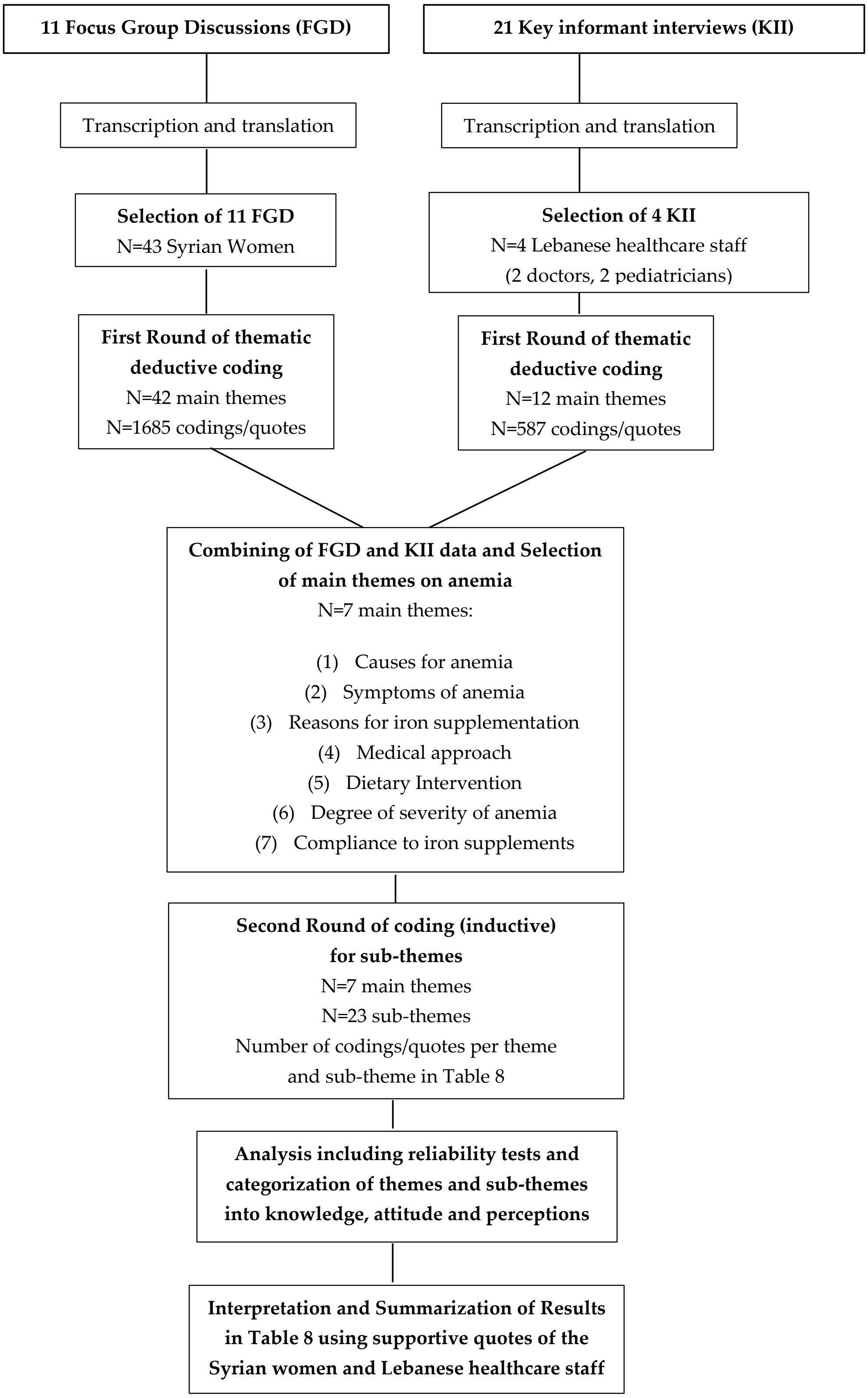

Table S2. Coding guidelines of main themes.

MAIN THEME	DEFINITION	ANCHOR SAMPLES	ENCODING RULES
**IRON PILLS CHILDREN**	Passages, that refer to iron pill supplementation of children	*"For the iron supplement that we're receiving from Ministry of Public Health. They try that many times and it was not that effective."* *(Nurse 1)*	The listing of iron pill supplementation of children or the possibility of iron supplementation
**IRON PILLS WOMEN**	Passages, that refer to iron pill supplementation of women	*"And with the supplements just coming back, because we noticed that, of course, during pregnancy they should take the iron."* *(Doctor 1)*	Specific supplementation of iron to pregnant or lactating women
**COST OF IRON PILLS AND TESTS**	Prices of iron deficiency related therapies	*"All medications are provided by the Ministry of Public Health, it's free 100%."* *(Nurse 2)*	Qualitative and quantitative references of prices of iron supplements, laboratory tests and clinical examinations
**ANEMIA CHILDREN**	Anemia among children related to clinical examinations, laboratory results or specific statements of the participants	*"I do not have numbers, but I know it is frequent. But I do not have an exact number of the kids that have anemia. The Ministry of Health of Lebanon is doing a study (unclear). I do not know if you know about it.. Every child that is less than 1 year old, we do for him a CBC. And if he has anemia - low hemoglobin, we will continue to do ferritin and iron."* *(Doctor 2)*	The listing of all activities related to anemia treatment of children
**ANEMIA WOMEN**	Anemia among women related to clinical examination or specific statements about anemia of the participants	*"Because we notice that so many said they had anemia during pregnancy."* *(Doctor 1)*	The listing of pregnant and lactating women who show symptoms of anemia
**FOODS GOOD FOR ANEMIA**	Foods and nutritional habits with positive effects on anemia and anemia treatment	*"Fresh juice, not the supermarket one"* *(Doctor 1)*	Referring to foods that ameliorate anemia
**KNOWLEDGE ON ANEMIA**	Knowledge and practical handling of anemia, information and specific nutritional knowledge	*"I think what they want is a magical solution. This is why I am telling you that education is important..."* *(Doctor 2)*	Information about knowledge of anemia including specific nutritional knowledge regarding anemia
**SOURCE OF NUTRITION INFORMATION**	Reference of nutritional information and advices given to Syrian mothers	*"Even if they are poor, they visit me on a regular basis to take advices. Education..."* *(Doctor 2)*	Indication of willingness to seek out professional advice
**MENTALITY OF WOMEN**	Psychological and social aspects influencing the nutrition of Syrian refugees and approach of anemia	*"Here in this area, if they know we got something for free, they would all come"* *(Nurse 2)*	Indications of psychological and social aspects affecting diets of Syrian refugees and their children

Table S3. Coding guidelines of sub-themes.

SUB-THEMES	DEFINITION	ANCHOR SAMPLES	ENCODING RULES
**CAUSES**	Causes of anemia disease or iron deficiency listed by participants	*"Anemia we have to much cases of anemia because of diet insufficiency"* (Doctor 1)	The listing of all causes related to anemia disease or iron deficiency
**SYMPTOMS**	Anemia associated symptoms listed by participants	*"The manifestations have […] hair, fatigue, pallor, asthenia. They don't have the strength to do anything but sit down."* (Doctor 2)	The listing of all symptoms associated with anemia
**MEDICAL TREATMENT**	Medical treatment of anemia with clinical examinations, laboratory results and iron pill prescription	*„With blood test[s] […] it shows hemoglobin hematocrit and MCV. So if I find any abnormal finding I make more blood test. For iron, for thalassemia"* (Doctor 1)	References to recommended medical treatments
**DIETARY INTERVENTION**	Adjustment of diet to either support iron pill treatment or to substitute the medical treatment	*"Every time one come in also I give iron supplementation, I advise them "do not give tea"."* (Doctor 1)	The listing of food to improve anemia or anemia treatment
**NO REACTION**	No reported change in behavior of Syrian mothers after diagnosis of anemia	*"Nothing is wrong with them […] well, my daughter's food intake is too minimal"* (Mother 10)	Statements about behavior include dietary and medical approach in response to anemia
**DEGREE OF SEVERITY**	Estimated level of severity and approach of anemia by participants	*„Anemia, we have to much cases of anemia"* (Doctor 1)	Qualitative classification of severity of anemia
**COMPLIANCE ANEMIA TREATMENT**	Psychological and social aspects influencing and interacting with the compliance of mothers	*"So, some of the mothers they will give up and they will not continue the treatment."* (Nurse 1)	Statements about compliance of anemia treatment as a whole
**REASONS**	Reasons listed by participants for iron pill supplementation of children and women	*"All babies who have IUGR and premature. We have to start iron because they don't have storage any from fetal life."* (Doctor 1)	The listing of reasons for iron pill prescription
**HEALTH SIDE EFFECTS**	Health side effects that emerge during and after iron pill intake	*"Side effects... stomach symptoms, maldigestion symptoms"* (Doctor 2)	The listing of health side effects occurring due to anemia treatment
**HEALTH IMPROVEMENT**	Significant improvement of anemia through iron pill supplementation	*"Those are for the kids you're talking about, there's a lot of improvement using them."* (Nurse 2)	Statements on recovery or improvement after iron pill supplementation
**COMPLIANCE IRON SUPPLEMENTATION**	Aspects that influence the practicability of iron pills and therefore the compliance	*"Yes, it's the kids it's not accepted or easily taken the medication"* (Nurse 1)	Classifying statements about practicability of iron pills to treat anemia
**EXPENSIVE**	Statements about expensive iron pill supplements	*"Multivitamins are free, iron not ... supplementation for children is not free. Iron is not free. When you donate the iron, it's not free."* (Doctor 2)	Qualitative and quantitative references of expenses due to anemia treatment
**FREE/ NOT EXPENSIVE**	Statements about free iron pill supplements or the possibility of generous price reduction	*"All medications are provided by the Ministry of Public Health, it's free 100%"* (Nurse 2)	Qualitative and quantitative references
**AVAILABILITY**	Availability of iron pills at health centers, medical offices or pharmacies	*"We don't have iron here"* (Nurse 2)	Statements on availability of iron pills that are preferably efficient

Table S4. Additional variables tested as determinants of child anemia

Child Variables (N = 214)	Not anemic	Anemic	Child Anemia	
Nutrition & Health status	***(n = 124)***	***(n = 90)***	**cOR (95%CI)**	**aOR (95%CI)**
**Stunting**				
Stunted (L/HAZ < -2 SD)	8 (6.5)	11 (12.4)	2.05 (0.79; 5.31)	0.66 (0.14; 3.12)
Not stunted (L/HAZ ≥ -2 SD)	116 (93.5)	78 (87.6)	1	1
**Had fever the past 14 days**				
No	68 (54.8)	41 (45.6)	1	1
Yes	56 (45.2)	49 (54.4)	1.45 (0.84; 3.91)	0.51 (0.20; 1.29)
**Dietary intake (24-h DR)**				
**Dairy**				
No	18 (14.5)	26 (28.9)	2.39 (1.22; 4.71) *	1.76 (0.59; 5.25) [c]
Yes	106 (85.5)	64 (71.1)	1	1
**Animal-source foods (ASF)**				
No	12 (9.7)	18 (20.0)	1	1
Yes	112 (90.3)	72 (80.0)	0.43 (0.20; 0.94) *	0.97 (0.29; 3.29) [d]
**Zaatar** *				
No	108 (87.1)	86 (95.6)	3.19 (1.03; 9.88) *	2.55 (0.45; 14.52)
Yes	16 (12.9)	4 (4.4)	1	1
**Maternal Variables**				
**Maternal anemia (past)**				
Not anemic	34 (28.1)	34 (37.8)	1	1
Anemic	87 (71.9)	56 (62.2)	0.64 (0.36; 1.15)	0.88 (0.37; 2.09)
**Knowledge on specific symptom of anemia**				
Not knowing that dizziness is a symptom	45 (38.8)	41 (49.4)	1.54 (0.87; 2-72)	0.90 (0.40; 2.01)
Knowing that dizziness is a symptom	71 (61.2)	42 (50.6)	1	1
**Maternal source of information**				
**Social media/internet**				
No	92 (74.2)	75 (83.3)	1	1
Yes	32 (25.8)	15 (16.7)	0.58 (0.29; 1.14)	1.35 (0.45; 4.03)
**Nurse/midwife**				
No	118 (95.2)	89 (98.9)	1	1
Yes	6 (4.8)	1 (1.1)	0.22 (0.03; 1.87)	0.23 (0.02; 3.38)
**Socio-economic variables**				
**Mother's education level**				
No schooling/illiterate	16 (13.0)	14 (15.9)	1.47 (0.60; 3.59)	-
Primary, Intermediate school	70 (56.9)	52 (59.1)	1.25 (0.66; 2.37)	-
Secondary school and higher	37 (30.1)	22 (25.0)	1	
**Father's education level**				
No schooling/illiterate	19 (15.4)	18 (20.0)	2.84 (1.02; 7.94) *	1.57 (0.35; 7.08)
Primary, Intermediate school	80 (65.0)	64 (71.1)	2.40 (1.01; 5.70) *	1.81 (0.57; 5.79)
Secondary school and higher	24 (19.5)	8 (8.9)	1	1
**Number of children in the household**				
1 to 2	82 (66.1)	47 (52.8)	1	1
3+	42 (33.9)	42 (47.2)	1.75 (1.00; 3.05)	1.62 (0.65; 4.08)
**Number of people in the household**				
1 to 5	65 (53.3)	37 (41.1)	1	1
6 to 20	57 (46.7)	53 (58.9)	1.63 (0.94; 2.83)	2.27 (0.97; 5.36)
**Experienced food insecurity of mother**				
Food secure/mildly food insecure	65 (53.7)	34 (38.6)	1	1
Moderately/severely food insecure	56 (46.3)	54 (61.4)	1.84 (1.06; 3.22) *	1.59 (0.65; 3.88)

* Zaatar: dried thyme-sesame powder mix.
